# Genetic diversity and structure of *Musa balbisiana* populations in Vietnam and its implications for the conservation of banana crop wild relatives

**DOI:** 10.1371/journal.pone.0253255

**Published:** 2021-06-23

**Authors:** Arne Mertens, Yves Bawin, Samuel Vanden Abeele, Simon Kallow, Dang Toan Vu, Loan Thi Le, Tuong Dang Vu, Rony Swennen, Filip Vandelook, Bart Panis, Steven B. Janssens

**Affiliations:** 1 Laboratory of Tropical Crop Improvement, Department of Biosystems, KU Leuven, Leuven, Belgium; 2 Meise Botanic Garden, Meise, Belgium; 3 Ecology, Evolution and Biodiversity Conservation, Department of Biology, KU Leuven, Leuven, Belgium; 4 Royal Botanic Gardens Kew, Millennium Seed Bank, West Sussex, United Kingdom; 5 Research Planning and International Department, Plant Resources Center, VAAS, Hanoi, Vietnam; 6 Department of Genebank Management, Plant Resources Center, VAAS, Hanoi, Vietnam; 7 International Institute of Tropical Agriculture, Arusha, Tanzania; 8 Bioversity International, Leuven, Belgium; 9 Molecular Biotechnology of Plants and Micro-organisms, Department of Biology, KU Leuven, Leuven, Belgium; Washington University, UNITED STATES

## Abstract

Crop wild relatives (CWR) are an indispensable source of alleles to improve desired traits in related crops. While knowledge on the genetic diversity of CWR can facilitate breeding and conservation strategies, it has poorly been assessed. Cultivated bananas are a major part of the diet and income of hundreds of millions of people and can be considered as one of the most important fruits worldwide. Here, we assessed the genetic diversity and structure of *Musa balbisiana*, an important CWR of plantains, dessert and cooking bananas. *Musa balbisiana* has its origin in subtropical and tropical broadleaf forests of northern Indo-Burma. This includes a large part of northern Vietnam where until now, no populations have been sampled. We screened the genetic variation and structure present within and between 17 Vietnamese populations and six from China using 18 polymorphic SSR markers. Relatively high variation was found in populations from China and central Vietnam. Populations from northern Vietnam showed varying levels of genetic variation, with low variation in populations near the Red River. Low genetic variation was found in populations of southern Vietnam. Analyses of population structure revealed that populations of northern Vietnam formed a distinct genetic cluster from populations sampled in China. Together with populations of central Vietnam, populations from northern Vietnam could be subdivided into five clusters, likely caused by mountain ranges and connected river systems. We propose that populations sampled in central Vietnam and on the western side of the Hoang Lien Son mountain range in northern Vietnam belong to the native distribution area and should be prioritised for conservation. Southern range edge populations in central Vietnam had especially high genetic diversity, with a high number of unique alleles and might be connected with core populations in northern Laos and southwest China. Southern Vietnamese populations are considered imported and not native.

## Introduction

Crop wild relatives (CWR) are wild species closely related to cultivated plants, including all direct progenitors of crops. Because of their relatedness, CWR provide useful trait characteristics for crop improvement such as stress tolerance and yield increase [1]. CWR are believed to become even more important for breeding in the near future due to climate change and increasing food demand [2,3]. A changing global climate may exacerbate stress levels on locally grown crops, whereas a rapidly expanding world population and changing dietary habits put a high pressure on food supplies [4,5]. Conservation of CWR in gene banks improves the accessibility of CWR to breeders. Nowadays, highly advanced genomic methods allow faster access to these genetic resources, and hence gene banks become even more valuable [6,7].

With an estimated production of 158.4 million tonnes in 2019, bananas (74%) and plantains (26%) can be considered as one of the most important fruits and staple foods of the world [8]. Although bananas are a main export product of many countries [9], 85% of the total production is consumed locally or sold regionally by producers [10]. Over one thousand existing varieties significantly contribute as a staple food to the daily diet and monthly income of hundreds of millions of people in developing regions in the subtropics and the tropics [11,12]. Next to the high caloric intake of the fruits, other parts of the plant can be used either as food, animal fodder, fibre, or medicine [13–15]. Despite the striking number of cultivars with different ploidy levels that have been described [16–19], almost all are derived from hybridization events between two species: *Musa acuminata* Colla (the so-called “A” genome) and *Musa balbisiana* Colla (“B” genome). Only a small number of cultivars contains genetic information of other *Musa* species such as *Musa schizocarpa* Simmonds and members of the former *Australimusa* section [20–23].

*Musa balbisiana* is a diploid (2n = 22) wild perennial herb of the “*Musa*” (former *Eumusa*) section of the genus *Musa* [24]. Its native distribution area ranges from northeast India to south China and northern Vietnam [25]. The presence of *M*. *balbisiana* on the Ryukyu islands, the Philippines, and Papua New Guinea is assumed to be feral after the introduction of this species by humans for cultivation purposes [26]. Asexual reproduction typically takes place close to the parent plant through suckers emerging from a lateral rhizome bud [27]. Inflorescences are supported by a pendulous stalk (also called peduncle) emerging from the pseudostem. Basal flowers are typically female with staminodes of varying levels of fertility, while male flowers are found at the tip of the inflorescence [28]. The flowers are pollinated by fruit bats, sunbirds, and likely a set of insects [29,30]. Seeds are dispersed by small foraging rodents [29].

*M*. *balbisiana* (BB) is one of the ancestors of diploid (AB), triploid (AAB, ABB), and tetraploid (ABBB, AABB, AAAB) plantains, cooking, and dessert bananas [17,31,32]. Note that the proportion of the A and B genome often deviates from the expected 1:1 ratio in the members of cultivar groups AB and AABB or from and expected 2:1 and 1:2 ratio in members of cultivar groups AAB and ABB respectively. This bias was recently attributed to interspecific recombination and large structural variations between the A and B genomes, highlighting a complex origin of cultivated bananas with one or multiple backcrosses [33–36]. In contrast to cultivars only consisting of genetic information of *Musa acuminata* (AA, AAA, AAAA), the presence of the B genome has been associated with increased drought and cold tolerance [37–40], resistance to *Xanthomonas* wilt [41–43], and tolerance against banana weevils [44]. The availability of a high quality reference genome [45] and recent identification of large structural variations between A and B genomes [36] will aid in the development of more optimal breeding of banana cultivars and considerable research has been done on assessing the geographic distribution and genetic diversity of wild *M*. *balbisiana* populations [29,46–49]. Still, wild populations of the species in their native distribution range are still underexplored, under-conserved, and potentially underused in breeding programs [7,50–53].

To date, only a few studies have investigated the intraspecific genetic diversity within *M*. *balbisiana*. Using Amplified Fragment Length Polymorphism (AFLP) markers, Ude et al. [54] assessed genetic diversity in eight *M*. *balbisiana* accessions obtained from gene banks and field collections. Their study was of the first to detect a high level of genetic diversity among *M*. *balbisiana* accessions. Ge et al. [29] and Wang et al. [49] found high levels of genetic diversity in fifteen Chinese *M*. *balbisiana* populations using AFLP and Simple Sequence Repeat (SSR) markers. The highest heterozygosity levels were observed in the Yunnan province, close to the Vietnamese border. Recently, Bawin et al. [46] used 18 SSR markers and demonstrated that seed batches sampled from additional populations of the Yunnan province in China harboured higher levels of genetic variation compared to more feral populations in Hainan (China), Amami (Japan), and Lae (Papua New Guinea), and to *ex situ* seed collections in Arusha (Tanzania) and Kampala (Uganda).

Species are often assumed to have larger population sizes, high genetic diversity, high gene flow and low differentiation in the centre of their distribution range [55]. In contrast, populations at the edge of their distribution range are generally believed to be more fragmented, smaller, and exhibit lower genetic diversity and higher genetic differentiation, though several studies report the lack of this latitudinal trend (e.g. [56] and [57]). Edge populations are often subjected to biotic or abiotic environmental conditions not present in these core populations [55,58]. They may adapt in response to these conditions, being potentially interesting for crop improvement because of unique or a larger frequency of adaptive alleles. Therefore, range-edge populations should not be ignored when collecting germplasm for conservation. *Musa balbisiana* populations at the southern edge of their native distribution range (e.g. populations in eastern Myanmar, northern Laos or northern-central Vietnam) might be exceptionally interesting. Populations may hold specific alleles adapted to better cope with drought stress, interesting for cultivated material containing the B genome, especially in a changing climate.

In this study, we for the first time quantify the genetic variation in Vietnamese populations of the banana crop wild relative *Musa balbisiana*. More specifically, we assessed the genetic structure and diversity in 17 Vietnamese *M*. *balbisiana* populations using 18 SSR markers. As large parts of northern Vietnam belong to the putative region of origin of the species, populations are expected to hold levels of genetic variation similar to those found in populations sampled in China. In addition, genetic variation in 6 wild populations of neighbouring Chinese provinces are compared with the Vietnamese populations to allow us to better understand the native character of the Vietnamese populations. Four questions are addressed: (1) How does genetic variation in Vietnamese *M*. *balbisiana* populations relate to that in Chinese populations? (2) How does genetic diversity vary among Vietnamese *M*. *balbisiana* populations? (3) Are *M*. *balbisiana* populations in Vietnam genetically structured along a geographic gradient? (4) Can southern populations of Vietnam be considered as wild?

## Materials and methods

### Taxon sampling

**Fig 1:**
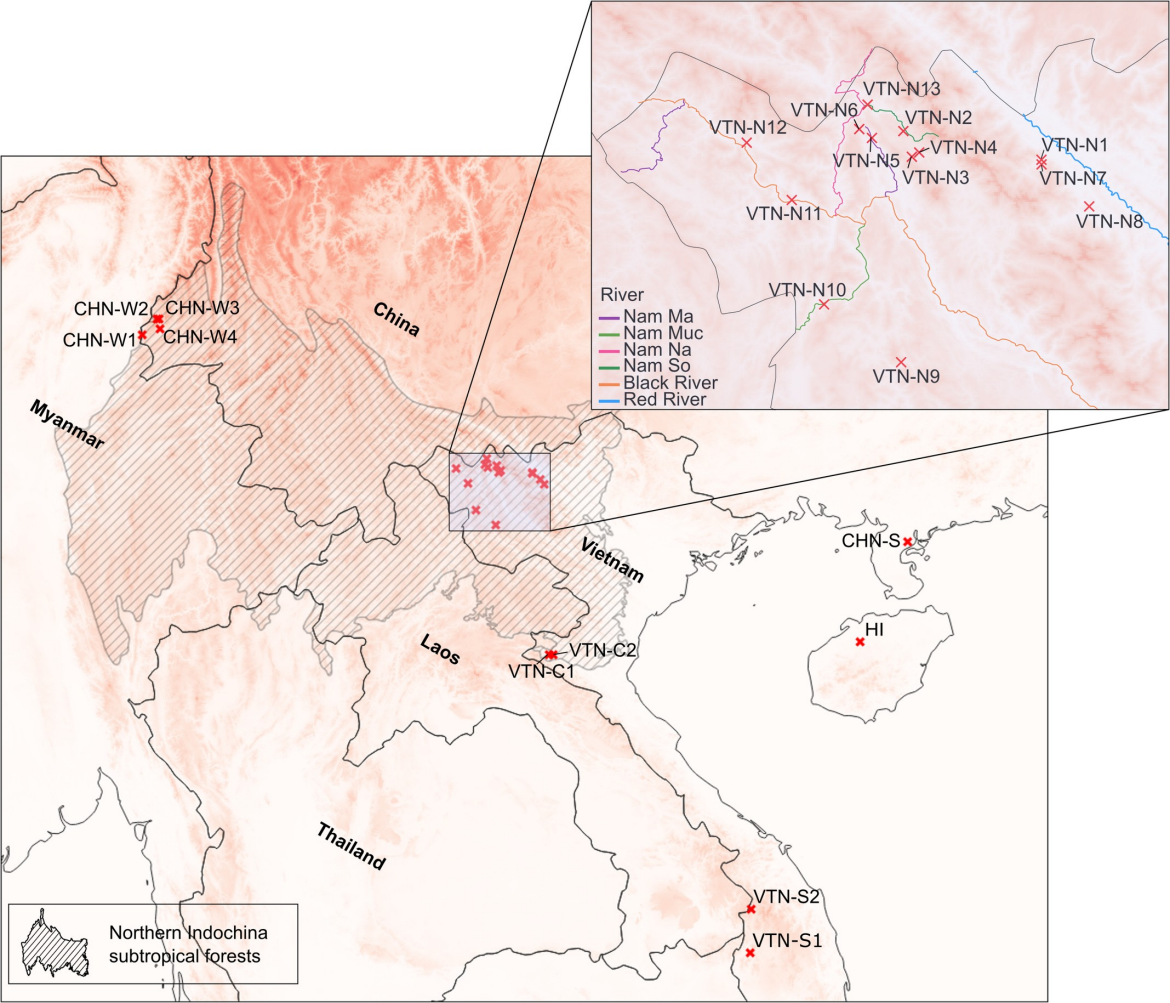
Sampling locations of wild *Musa balbisiana* populations. Shaded area represents the northern Indochina subtropical forests, the native distribution area of *M*. *balbisiana*. Geospatial datasets used for the creation of these maps were reprinted from [59–61] under a CC-BY 4.0 license.

Field research was carried out as a collaboration between Meise Botanic Garden (Belgium) and the Plant Resources Center (Hanoi, Vietnam). The Plant Resources Center had permission for working with Meise Botanic Garden under the Bilateral cooperation project: the Vietnamese National Foundation for Science and Technology Development (NAFOSTED). Field permits for Yen Bai (564/UBND-NV), Ha Giang (255/SNN-CCLN), and Lao Cai (1060/UBND-NC) were obtained. During multiple collection missions carried out between 2017 and 2020, 274 individuals belonging to 17 populations of wild *Musa balbisiana* were sampled in Vietnam (Table 1, Fig 1). Populations were selected along a north-south geographic gradient ranging between a latitude of 22.39 and 14.37 a longitude of 102.78 and 107.68. Whenever possible, leaves from a minimum of 15 individuals per population were sampled. In order to avoid sampling suckers from the same individual, plants located directly next to each other were not sampled. In addition to leaf material from Vietnam, six seed collections of wild *M*. *balbisiana* from China were also included in this study. Three seed collections (two from Yunnan and one from Guangdong) were newly acquired and used in the present study. Finally, data from Bawin et al. [46] for three seed collections (two from Yunnan and one from Hainan) was also included in the analysis. Each seed collection was retrieved from one banana bunch per sampling location, a common practise in collecting banana seeds.

**Table 1:** Sampling location information.

Country	population code	Geographical name	N	Origin (province, locality)	Latitude	Longitude	Material type
**Vietnam**	VTN-N1	northern Vietnam 1	20	Lao Cai, Muong Cau	22.31417	104.0397	Leaf
**Vietnam**	VTN-N2	northern Vietnam 2	16	Lai Chau, Can Cau	22.43667	103.4497	Leaf
**Vietnam**	VTN-N3	northern Vietnam 3	11	Lai Chau, Cu Ti	22.32689	103.4861	Leaf
**Vietnam**	VTN-N4	northern Vietnam 4	20	Lai Chau, Na Bo	22.34528	103.5175	Leaf
**Vietnam**	VTN-N5	northern Vietnam 5	13	Lai Chau, Nam Ma Dao	22.4075	103.3156	Leaf
**Vietnam**	VTN-N6	northern Vietnam 6	14	Lai Chau, Seo Leng	22.44528	103.2614	Leaf
**Vietnam**	VTN-N7	northern Vietnam 7	13	Bao Thang, Suoi Thau	22.298	104.0424	Leaf
**Vietnam**	VTN-N8	northern Vietnam 8	12	Van Ban, Khanh Yen Thuong	22.11497	104.2439	Leaf
**Vietnam**	VTN-N9	northern Vietnam 9	16	Dien Bien, Xa Tu	21.44953	103.4403	Leaf
**Vietnam**	VTN-N10	northern Vietnam 10	21	Dien Bien, Na Sang	21.6965	103.1113	Leaf
**Vietnam**	VTN-N11	northern Vietnam 11	20	Lai Chau, Nam Hang	22.14219	102.9731	Leaf
**Vietnam**	VTN-N12	northern Vietnam 12	19	Lai Chau, Bum To	22.38722	102.7806	Leaf
**Vietnam**	VTN-N13	northern Vietnam 13	4	Lai Chau, Vang Bo	22.54954	103.297	Leaf
**Vietnam**	VTN-C1	central Vietnam 1	20	Nghe An, Ban Phung	19.29876	104.3213	Leaf
**Vietnam**	VTN-C2	central Vietnam 2	20	Nghe An, Khe Ngau	19.3028	104.3854	Leaf
**Vietnam**	VTN-S1	southern Vietnam 1	18	Kontum, Mo Rai	14.35633	107.6624	Leaf
**Vietnam**	VTN-S2	southern Vietnam 2	17	Kontum, Dak Nhoong	15.08056	107.6817	Leaf
**China**	CHN-W1	western China 1	19	Yunnan	24.60678	97.58322	Plants grown from seeds
**China**	CHN-W2	western China 2	20	Yunnan	24.86883	97.82441	Plants grown from seeds
**China**	CHN-W3	western China 3	14	Yunnan	24.87246	97.85518	seed
**China**	CHN-W4	western China 4	7	Yunnan	24.69901	97.87019	seed
**China**	CHN-S	southern China	11	Guangdong	21.1622	110.2756	seed
**China**	HI	Hainan	20	Hainan	19.51667	109.4833	Plants grown from seeds

N, number of included samples per population of wild *Musa balbisiana*.

### SSR genotyping

Banana leaf material was dried for preservation in the field using silica gel [62]. Seed embryos were excised from seed collections acquired in this study using a surgical scalpel and added to 10 μl of cetyltrimethylammonium bromide (CTAB). DNA from leaves and embryos was subsequently isolated using a modified CTAB extraction protocol of Doyle & Doyle [63]. In order to compare our data to the genetic variation observed in Bawin et al. [46], we used the same set of 18 microsatellite markers arranged in four multiplexes based on their polymorphic character from multiple studies assessing both wild and cultivated material (S1 Table). To infer the genomic coordinates from nuclear SSR markers, GenBank sequences retrieved from Wang et al. [64] and Rotchanapreeda et al. [65] of each marker were blasted against the reference genome sequence of *Musa balbisiana* “Pisang Klutuk Wulung” in the Banana genome hub [66]. An expect cutoff value of 1e-10 was used and the single best search result was retained. The genomic coordinates from this search were then plotted inside chromosome tracks in R [67].

PCR of microsatellite fragments was carried out with Qiagens Type-it Microsatellite PCR Kit (Qiagen, Venlo, the Netherlands). Subsequently, 1 μl of diluted PCR sample was added to 12 μl HiDi Formamide mixed with 0.4 μl of the MapMarker 500 labelled with DY-632 (Eurogentec, Seraing, Belgium), after which 1.5 μl of this product was genotyped on an ABI 3730 system (Applied Biosystems, Foster City, California) at the Université Libre de Bruxelles (ULB), Belgium. For more recent samples, 20 μl of PCR product was sent to Macrogen (Macrogen Europe, Amsterdam, the Netherlands) for genotyping on an ABI 3730 system. A minimum of ten samples per genotyping run were ran in duplicate to deal with potential differences in fragment sizing. Raw data were scored and checked for errors in Geneious Prime 2021.0.3 (Biomatters, New Zealand) with a third order least squares method. To ensure a uniform scoring of alleles, raw data obtained from the two populations of Yunnan and one from Hainan from the study of Bawin et al. [46] were re-scored using the same sizing method. Samples with ambiguous patterns were genotyped twice to resolve erroneous scoring, and samples with more than 10% missing data were excluded from the analyses. To screen our data for outliers, the GENETIX software [68] was used and one population was removed from the dataset (Lao Cai, Nam Ma commune) based on an AFC-3D visualisation of the genotypic data (S1 Fig).

### Genetic diversity indices

The average number of alleles per locus with a frequency larger than 5% (Na), the average number of private alleles unique to a single population (Np), Shannon’s Diversity Index (H), proportion of polymorphic loci (P), Unbiased expected Heterozygosity (uHe), and Observed Heterozygosity (Ho) were calculated with the GenAlEx 6.51 Excel Package [69]. Inbreeding coefficients were additionally calculated with the “fs.dosage” function in R package “hierfstat” [70].

### Population genetic structure

To explore our genetic data and to visualize the genetic structure of populations, we assessed the pairwise genetic differentiation between populations through codominant genotypic distances and used a Principal Coordinate Analysis (PCoA) as visualisation. With GenAlEx, an analysis of molecular variance (AMOVA) was run using 999 permutation steps to assess the genetic variation present within and between sampled populations while taking the sampled region into account. Here, genetic differentiation was measured as F_ST_. Within and between population variation including all samples was additionally visualised by a PCoA constructed using a Euclidean distance matrix with the “dudi.pco” function in R package “ade4” [71].

Population genetic structure was assessed using Bayesian clustering in STRUCTURE 2.3.4 [72]. All runs using STRUCTURE were done on the online Galaxy platform [73] using an admixture model to allow samples to be assigned to one or multiple genetic sources (clusters). Because unequal population sampling may lead to poor estimations of the optimal number of genetic clusters (K), we followed the methodology of Wang [74] and used the uncorrelated allele frequency model with a separate ancestry prior alpha (α) for each cluster while setting the initial α to 1/K.

First, the optimal number of genetic clusters to which individuals could be assigned to was determined by testing 10 independent runs for a K value ranging from one to 20 using a model with correlated allele frequencies and a default initial α of 1.00 for all clusters. For each independent run, 100,000 Markov Chain Monte Carlo (MCMC) iterations were sampled after a burn-in of 100,000 iterations. The optimal number of genetic clusters was then determined based on ΔK and the log posterior probability of the replicates over each K [75]. This value of K was additionally compared to the optimal number of clusters based on the MedMeaK (median of means), MaxMeaK (maximum of means), MedMedK (median of medians), and MaxMedK (maximum of medians) as described in Puechmaille [76]. These descriptors were calculated on the online web server of StructureSelector [77]. The second set of analyses was then run using the same parameter configurations, but this time allowing separate α values for each cluster and an initial α of 1/K, with a K inferred from the first run, with independent allele frequencies. Samples with a cluster assignment probability < 0.8 were considered to be admixed and were not assigned to one specific inferred cluster.

Two datasets were used to run the clustering analyses in STRUCTURE. The first dataset contained all samples from all populations after removing any outlying samples, allowing us to directly compare populations of Chinese with those of Vietnamese origin. The second dataset consisted of only Vietnamese populations in their native distribution range (northern Indochina). This allowed us to evaluate the population genetic (sub)structure of these populations. Structure plots with the optimal K were made with the CLUMPAK software as implemented in StructureSelector [78].

### Isolation by distance

To estimate whether genetic distance also increases when populations are geographically more distant (Isolation by distance), a geographic distance matrix was calculated based on the geographic coordinates of each population. This was done for the two different datasets, one including all sampled populations and one only including populations sampled in northern and central Vietnam. For both datasets, a paired Mantel test analysis was performed between the geographic and genetic distance matrices (Edward’s distance) with R package “ade4” to assess whether genetic isolation by distance was significant [71]. All R packages were used in R version 4.0.2 [67].

## Results

### Genomic coordinates

Blasting SSR marker sequences against the reference genome showed that they are spread across 8 out of 11 chromosomes (S2 Table). No markers covered chromosomes 1, 5, and 7 and some of the remaining chromosomes were covered by multiple markers. Plotting the geographic coordinates revealed that some markers were located in close proximity to each other and that large parts of chromosomes are not covered (S2 Fig).

### Genetic diversity

The average number of alleles per locus with a frequency larger than 5% was low in all sampled populations, ranging from 1.11 in populations VTN-N8 and VTN-S1 to 1.94 in VTN-C1 (Table 2). Fourteen populations did not contain private alleles, and the highest average number of private alleles was found in VTN-C1 and CHN-S. The Shannon diversity index was lowest in VTN-S1 and highest in VTN-N10. The proportion of polymorphic loci ranged from 11% to 67% and was low (<20%) in the southern populations (VTN-S1, VTN-S2) and some northern populations (VTN-N3, VTN-N8). Expected and observed levels of heterozygosity ranged between 0.05 to 0.28, with an average of 0.19 and 0.16 respectively, though strongly varied between populations. Low levels of inbreeding were found across all populations, but six *M*. *balbisiana* populations had an excess in heterozygotes (negative values) (VTN-N3, VTN-N8, VTN-N9, VTN-N13, VTN-S1, and CHN-W3).

**Table 2:** Intra-population genetic diversity indices of *Musa balbisiana* populations.

Pop	Na	Np	H	P (%)	H_e_	H_o_	F_IS_
**VTN-N1**	1.222	0.000	0.085	0.222	0.054	0.056	-0.015
**VTN-N2**	1.556	0.000	0.253	0.389	0.157	0.147	0.054
**VTN-N3**	1.167	0.000	0.115	0.167	0.087	0.152	-0.818
**VTN-N4**	1.500	0.056	0.263	0.556	0.169	0.172	-0.020
**VTN-N5**	1.722	0.000	0.323	0.500	0.208	0.218	-0.048
**VTN-N6**	1.611	0.000	0.331	0.444	0.218	0.218	-0.001
**VTN-N7**	1.222	0.111	0.101	0.222	0.067	0.051	0.247
**VTN-N8**	1.111	0.000	0.077	0.111	0.058	0.111	-0.968
**VTN-N9**	1.278	0.000	0.193	0.278	0.143	0.278	-1.000
**VTN-N10**	1.889	0.111	0.427	0.444	0.250	0.257	-0.026
**VTN-N11**	1.778	0.000	0.331	0.500	0.200	0.206	-0.027
**VTN-N12**	1.722	0.000	0.330	0.444	0.204	0.202	0.011
**VTN-N13**	1.278	0.000	0.193	0.278	0.159	0.278	-1.000
**VTN-C1**	1.944	0.056	0.401	0.556	0.250	0.269	-0.078
**VTN-C2**	1.889	0.222	0.423	0.667	0.254	0.256	-0.005
**VTN-S1**	1.111	0.000	0.075	0.111	0.055	0.080	-0.482
**VTN-S2**	1.167	0.056	0.109	0.167	0.079	0.085	-0.030
**CHN-W1**	1.778	0.056	0.372	0.611	0.233	0.241	-0.064
**CHN-W2**	1.556	0.000	0.285	0.500	0.171	0.183	-0.078
**CHN-W3**	1.278	0.056	0.153	0.333	0.106	0.175	-0.697
**CHN-W4**	1.778	0.000	0.358	0.556	0.243	0.243	0.012
**CHN-S**	1.556	0.167	0.319	0.444	0.223	0.210	0.067
**HI**	1.556	0.000	0.251	0.444	0.152	0.161	-0.061

Na, number of different alleles with frequency >5%; Np, average number of private alleles unique to a single population; H, Shannon’s Diversity Index; P (%), proportion of polymorphic loci; H_e_, expected heterozygosity; H_o_, observed heterozygosity; F_IS_, inbreeding coefficient.

### Genetic structure

**Fig 2:**
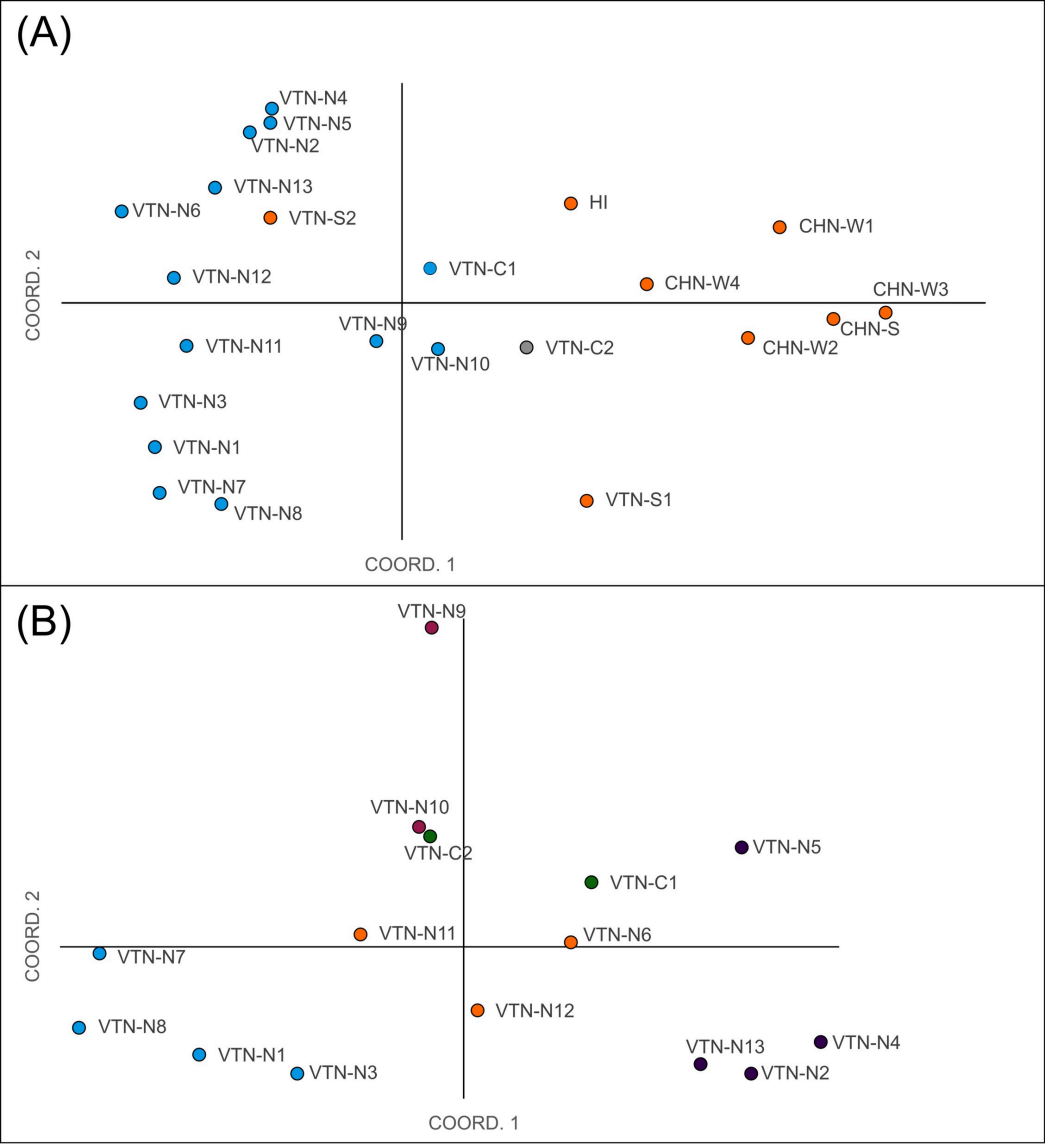
Principal coordinate analysis of wild populations of *Musa balbisiana* based on a Codom genotypic genetic distance matrix. (A), full dataset; (B), Native Vietnamese populations. Colours are based on cluster assignments of the STRUCTURE analyses below (see Fig 3).

In the Principal Coordinate Analysis of the full dataset, the first two axes explained together 26.6% and 17.8% of the genetic variation present between the populations respectively (Fig 2A). The Chinese and northern Vietnamese populations were separated from each other in the PCoA plot, whereas additional sub-clustering was observed within Vietnamese populations. VTN-S1 did not clearly group with any of the other populations. The AMOVA showed that the among population variation was lower (32%) in comparison with the genetic variation present among individuals within different populations (53%). Fifteen percent of the observed genetic variation was attributed to the sampling regions (S3 Fig). This is also evident from the PCoA with all samples included (S4 Fig). In the dataset containing only Vietnamese populations in northern Indo-Burma (VTN-N and VTN-C), the first two axes of a PCoA explained together 35.6% and 27.9% of the genetic variation respectively (Fig 2B). Some population structuring was found. Populations VTN-N1, VTN-N3, VTN-N7, and VTN-N8 were grouped together as well as VTN-N2, VTN-N4, and VTN-N13. VTN-N9 was more distant to the other sampled populations.

**Fig 3:**
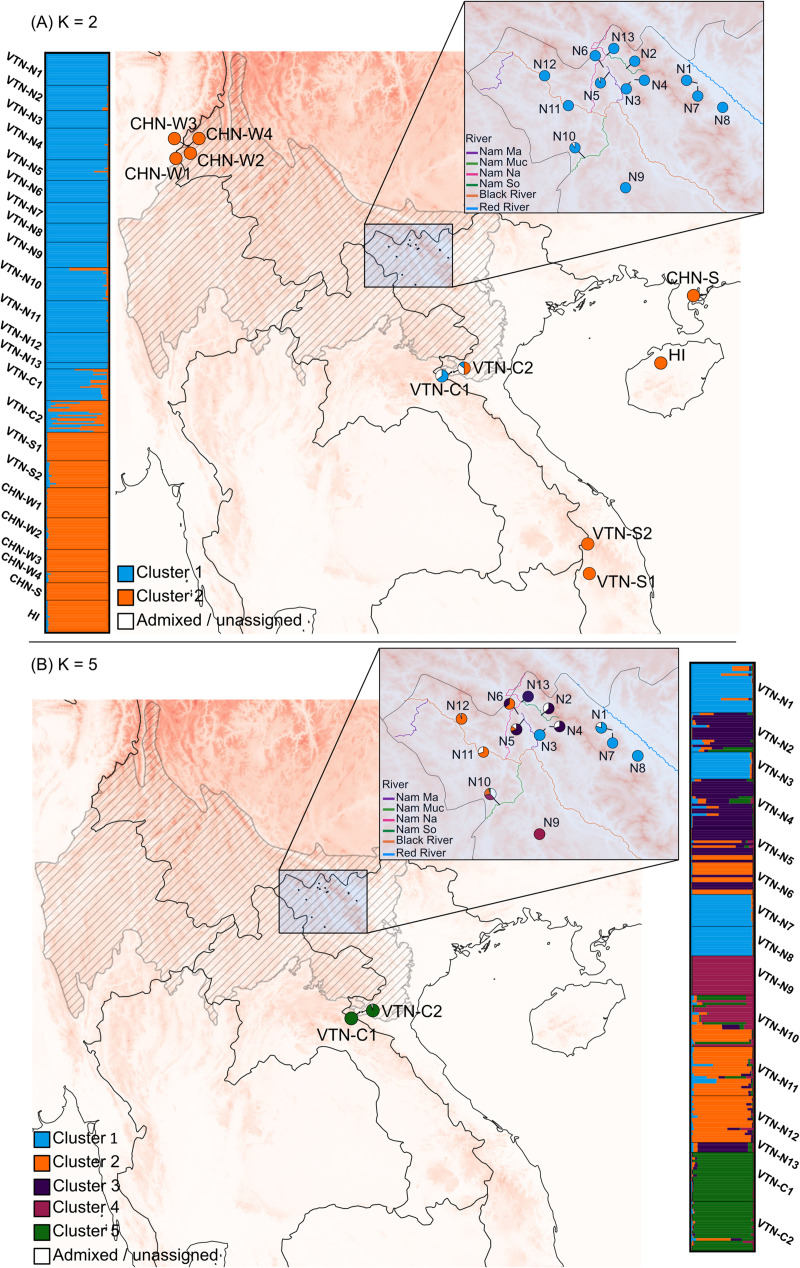
Bar plots indicating cluster assignments obtained with STRUCTURE. Bar plots are presented for the full dataset (A) and the reduced dataset only including native Vietnamese populations (B) with K = 2 and K = 5, respectively. Pie charts indicate cluster assignment values of individuals within each population. White pieces indicate the partition of individuals that could not be assigned to one specific cluster, using an assignment probability threshold of 0.8. Geospatial datasets used for the creation of these maps were reprinted from [59–61] under a CC-BY 4.0 license.

In both sets of genetic clustering analyses using STRUCTURE with correlated allele frequencies, the optimal number of clusters was different for the three assessment criteria, making it hard to determine the optimal number of clusters (S3 Table). Because the high optimal number of clusters suggested by most criteria (K ranging from 11 to 19) were very hard to interpret, we considered the Evanno method (ΔK/K) to assess the number of clusters. For both datasets, this resulted in an optimal number of K = 2 clusters (S5 Fig). However, it has been shown that the Evanno method is often biased towards K = 2 as the most probable number of genetic clusters [79]. Therefore, population stratification was also assessed using the second-highest value for ΔK/K and by comparing the STRUCTURE results for all K values to the PCoA. For the full dataset, no K value lower than K = 16 was found to confidently represent the population structure thus K = 2 was considered to be most optimal, which was also supported by the PCoA (Fig 2A). For the dataset with only native Vietnamese populations, K = 5 was considered most reliable based on this method and the PCoA (Fig 2B). These K values were used to run the final set of analyses with uncorrelated allele frequencies with a separate α for each cluster and with an initial α = 0.5 and α = 0.2 for the full and restricted analyses, respectively, using independent allele frequencies. In the full analysis (K = 2), only 17 individuals had an assignment probability of < 0.8 and were therefore not assigned to a specific cluster. Most of them were individuals from populations sampled in central Vietnam (VTN-C1 and VTN-C2). Almost all individuals from native populations from Vietnam (except some from VTN-C2) were assigned to the same cluster, while individuals from Chinese populations and from populations sampled in south Vietnam were all assigned to the other cluster (Fig 3A). Using a K = 5 for the native Vietnamese populations resulted in 35 individuals from 9 different populations with an assignment probability to a specific cluster of < 0.8. Most individuals of four populations (VTN-N1, VTN-N3, VTN-N7, VTN-N8) were mainly assigned to cluster 1, most of three other populations to cluster 2 (VTN-N6, VTN-N11, VTN-N12), and individuals of another four populations to cluster 3 (VTN-N2, VTN-N4, VTN-N5, VTN-N13). All individuals from VTN-N9 were assigned to their own cluster 4. Plants from central Vietnam were almost all assigned to cluster 5. Some individuals of VTN-N10 were assigned to clusters 1, 2, or 4, but most individuals of this population could not be assigned to a specific cluster (Fig 3B).

**Fig 4:**
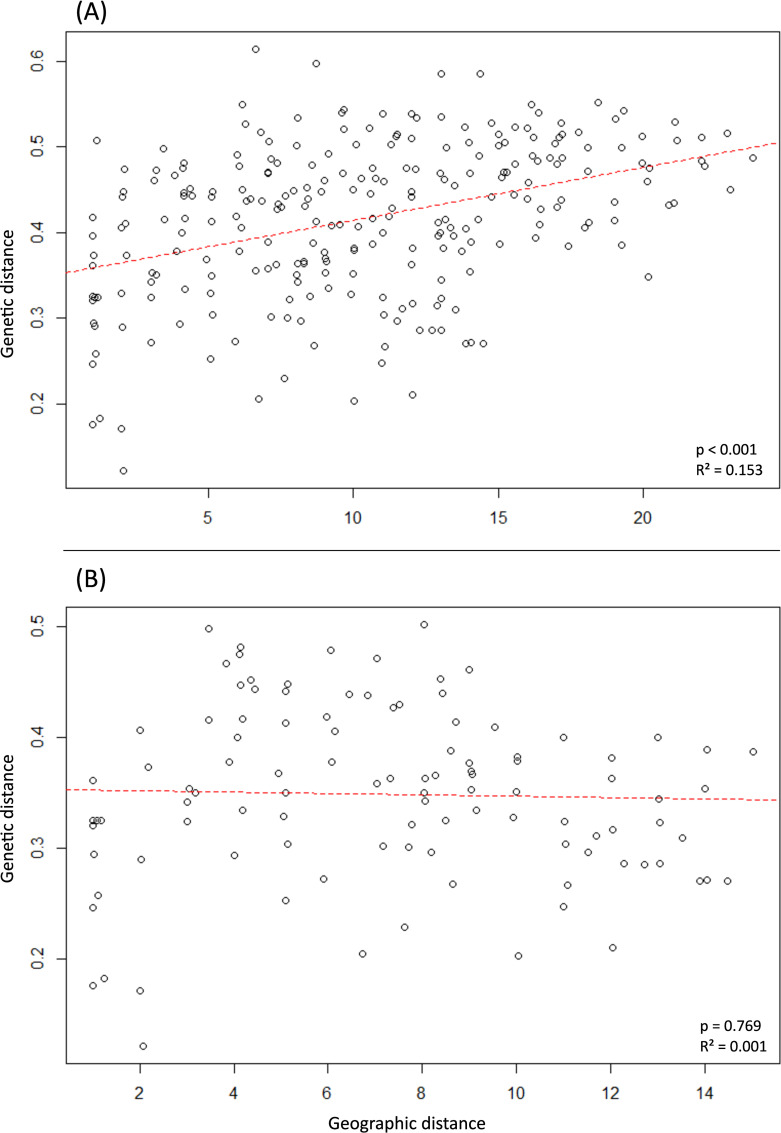
Isolation by distance plot. Geographic distance is displayed on the x-axis and genetic distance on the y-axis for (A) the complete dataset, and (B) the reduced dataset including only native Vietnamese populations.

Mantel tests between geographic and genetic distances revealed a significant pattern of genetic isolation by distance between wild *M*. *balbisiana* populations (p < 0.01) when assessing all populations. However, the regression curve only accounted for 15% of the observed response (R^2^ value of 0.153) (Fig 4A). When assessing only the Vietnamese populations, no pattern of IBD was found (p = 0.769) (Fig 4B).

## Discussion

### Genetic diversity in *Musa balbisiana* populations

In this study, we assessed for the first time the genetic variation of Vietnamese populations of *Musa balbisiana* and complemented them with populations sampled in the Yunnan and Guangdong province of China, as well as a population of Hainan island. For this, we used a set of 18 polymorphic microsatellite markers that were combined and in four multiplex PCRs in a previous study [46]. This allowed us to compare genetic diversity of Vietnamese populations with populations sampled in the regions that have been suggested to be part of the native distribution region of *M*. *balbisiana* [29,49]. Blasting the markers for which the sequences were available against the reference genome showed that most chromosomes were covered. However, some were only covered by one marker and large parts of chromosomes are not optimally covered using SSRs. Still, compared to the 11 out of 18 markers Bawin et al. [46] found to be polymorphic, an additional two (13 in total) were polymorphic for our dataset, validating that these markers can detect sufficient genetic variation. While a direct comparison is not possible with the populations used in these studies due to differences in genetic markers (AFLP or other SSR markers) and incomplete information on data processing, general patterns in genetic diversity can be compared. In relation to studies that similarly assess the genetic variation of populations of other CWR species using SSRs (e.g. [80–82]), the genetic variation we found in all *M*. *balbisiana* populations was rather low, with observed heterozygosity levels ranging from 0.05 to 0.28 with an average of 0.19. Low genetic variation in populations can be expected in small, fragmented populations or with low gene flow between populations and high levels of inbreeding [83]. Comparable to population genetic studies of *M*. *balbisiana* and other crop species with clonal reproduction capacities [84,85], we found negative inbreeding coefficients (heterozygotic excess) in six populations and overall high levels of within population variation (53%) versus among population variation (32%). In bananas, excess of heterozygosity could be explained mainly by three mechanisms. First, while the species is not self-incompatible, selfing is limited in *M*. *balbisiana* due to the way the flowers develop. The flowers at the base of the inflorescence are typically female, followed by hermaphrodite flowers with aborted female and male organs at the tip of the inflorescence. Due to different flowering times, self-pollination is largely prohibited. Second, the species can propagate clonally. Clonal reproduction might result in a heterozygosity excess because of a low number of genotypes present in a population (low expected heterozygosity) while maintaining heterozygosity for a large proportion of the assessed loci [86]. Lastly, levels of heterozygosity could be maintained through the accumulation of somatic mutations over long periods of time, but it is very unlikely that, if this is the case, can be picked up with 18 SSR loci [29,84,85,87].

Despite the relatively low observed levels of genetic variation, clear differences were found between populations. Excluding the population from Hainan, Chinese populations on average had moderate to high percentages of polymorphic loci, expected and observed levels of heterozygosity as well as low levels of inbreeding. In previous studies on genetic diversity in Chinese *M*. *balbisiana* populations, high relative levels of genetic diversity were also found in populations sampled in Yunnan, Guanxi and southern Guangdong, especially for the population of Yunnan in the northern Indochina subtropical region [29,49]. They suggest that the high genetic diversity in the more western Chinese *M*. *balbisiana* populations is potentially caused by the presence of long-tongued fruit bats (*Macroglossus sobrinus*) that act as a long-distance pollinator in western China.

In contrast to the relatively high genetic variation observed in Chinese populations (H_o_ ranging from 0.175 to 0.243), the genetic diversity in Vietnamese *Musa balbisiana* showed larger differences between populations (H_o_ ranging from 0.051–0.278). Populations sampled in south Vietnam, near the Red River Basin (VTN-N1, VTN-N7, VTN-N8) as well as VTN-N3 in Lai Chau had low genetic diversity, with a low number of different alleles, a limited number of private alleles, a low number of polymorphic loci, and low heterozygosity levels. Three populations had high levels of these indices and low levels of inbreeding (VTN-N10, VTN-C1, VTN-C2), while the other populations had moderate levels. Most notable is that the populations of central Vietnam (VTN-C1, VTN-C2) had the highest levels of different alleles and number of polymorphic loci and in particular VTN-C2, which had the highest proportion of private alleles compared to the other populations. Compared to the Chinese populations, similar levels of genetic diversity were found in northern and central Vietnam, excluding VTN-N1, VTN-N3, VTN-N7, and VTN-N8. Noteworthy, according to the bats checklist of Vietnam [88], *Macroglossus sobrinus* has only been recorded in the close vicinity of those central Vietnamese *M*. *balbisiana* populations (VTN-C1,VTN-C2). However, other sources also report *M*. *sobrinus* bats further North (including where VTN-N9 and VTN-N10 occurred) [89].

In the PCoA considering all sampled populations, Vietnamese populations could clearly be distinguished from Chinese populations (Fig 2A). This was supported by STRUCTURE analyses, with the exception of high admixture between the two clusters in VTN-C2 and populations of southern Vietnam clustering with Chinese populations. We did not observe a pattern of isolation by distance in the northern and central Vietnamese populations, and only a low (though significant) R^2^ value of the IBD pattern for all assessed populations. This can be caused by either limited gene flow due to geographic barriers or a lack of suitable long-distance pollinators on the one hand, or excessive gene flow homogenising genetic variation among populations on the other hand. The former has been linked with a potential decline in fruit bats due to habitat loss, bushmeat hunting, and climate change [49,90]. Because bananas can easily propagate via suckers, a long-distance seed dispersal event might result in the establishment of a healthy population genetically similar to the source population, but with no or limited gene flow after colonisation. Apart from VTN-N3 from Lai Chau and the populations near the Red River (VTN-N1, VTN-N7, VTN-N8), northern and central *M*. *balbisiana* populations can be considered to be a part of the native distribution area of the species based on their levels of observed heterozygosity. However, the origin of the two remote *M*. *balbisiana* populations located in southern Vietnam (Kon Tum province) remains more uncertain. Populations in southern Vietnam might be more connected to *M*. *balbisiana* populations in Laos and Cambodia than to other populations in Vietnam, which may explain the genetic differentiation of the southern populations from other Vietnamese populations included in this study. Nevertheless, the distribution of *M*. *balbisiana* in these neighboring countries is obscure, impeding the inference of any potential link with the populations in southern Vietnam. Alternatively, the southern *M*. *balbisiana* might be feral, originating after the recent human introduction of the species in the region as a source of food, medicine, fibre, and animal fodder. *M*. *balbisiana* was known by locals in northern Vietnam because of its higher tolerance to cold, drought and potential plant diseases, encouraging its cultivation in other parts of the country [38,39,42]. The southern populations of *M*. *balbisiana*, especially VTN-S1, were also found close to cultivated varieties. Moreover, these populations were located far outside the range of the northern Indo-Burma subtropical forests, the native habitat of the other Vietnamese *M*. *balbisiana* populations. Taken together and in absence of information on *M*. *balbisiana* in neighboring countries, southern Vietnamese populations of *M*. *balbisiana* are supposedly no part of the native distribution area of the species.

### Genetic structure of native Vietnamese *Musa balbisiana*

By inferring genetic clusters solely among the Vietnamese populations that occur in the northern Indochina subtropical forests, the northern and central Vietnamese *M*. *balbisiana* populations could be subdivided into five different groups. This result was supported by both the PCoA and the STRUCTURE analyses. Interestingly, the genetic diversity indices followed to a large extent this subdivision. In this section, we discuss the observed patterns of genetic structure among the northern and central Vietnamese *M*. *balbisiana* populations.

In northern Vietnam, four populations (VTN-N1, VTN-N3, VTN-N7, VTN-N8; Fig 3B) clearly grouped together in all analyses, especially in the STRUCTURE analysis. They shared very low levels of genetic diversity. These populations, apart from VTN-N3 sampled in Lai Chau, were located between the east side of the Hoang Lien Son mountain range and the Red River, and showed limited genetic admixture with the other clusters. The northwest-southeast orientation of the Red River valley separated these populations from the northeastern montane regions. This suggests that the Red River acted as geographic barrier that prevents gene flow between populations in the east, a pattern that has been observed in two frog species [91] and a species of *Cycas* [92]. As a southern extension of the Himalayas with multiple peaks above 2,500m and even up to 3,143m on Fansipan mountain, the Hoang Lien Son mountain range might act as a barrier to the west [93]. How and why VTN-N3 was assigned to this cluster is uncertain. We propose that this might be the result of a one-time long dispersal event that was mediated by either animals or humans, with limited connectivity to neighbouring populations. Another hypothesis is that all populations belonging to cluster 1 have a history of human influence. For example, *M*. *balbisiana* is known to be introduced from China to the Ryuku isles in Japan already in the 16^th^ century for fiber use, making it hard to consider all sampled populations as wild, especially when they have low genetic variation and are located close to cultivated areas [26].

On the western side of the Hoang Lien Son mountain range, populations VTN-N2, VTN-N4, VTN-N5, and VTN-N13 (cluster 2, Fig 3) are geographically close to each other and follow to some extent a small river complex (Nam So, Nam Na, Nam Ma, Fig 1). The link between populations of cluster 3 and the Black River (Song Da, Fig 1) is clear, with a direct connection between VTN-N11 and VTN-N12 and an indirect connection with VTN-N6 via the Nam Na river that also connects to some extent to the Nam so. This could potentially explain the assignment of some individuals of VTN-N6 to either cluster 2 or cluster 3. The Nam Muc river indirectly connects the highly admixed VTN-N10 to the Black River system. The high genetic diversity found in this population might be due its proximity to VTN-N9 that was assigned to its own cluster as well to the Black River system or to populations in Laos. Though all individuals from VTN-N9 were assigned to its own cluster, this population had a relatively high level of observed heterozygosity and an inbreeding coefficient of -1, suggesting that this population might be for a large part reproducing clonally [87,94]. The link between river systems and genetic clustering could be the heterogenous topography of northwestern Vietnam with peaks over 2,000m a.s.l. that can act as a physical barrier for both seed dispersal and pollination by bats and insects via land [95,96].

Populations sampled in central Vietnam showed the highest number of different alleles, the highest proportion of private alleles and the highest percentage of polymorphic loci. Both populations also had among the highest levels of heterozygosity of all sampled Vietnamese populations. Both populations clearly clustered together in the STRUCTURE analyses. Geographically, they can be considered to be at the southern range edge of the northern Indo-Burmese region. In theory, populations at the edge of the distribution range are expected to be more isolated and smaller, with lower levels of genetic diversity within populations but higher genetic differentiation between populations due to reduced gene flow [55]. This can result in high levels of regional diversity at the range edge populations, hence worth preserving. Moreover, local adaptations and unique alleles present in these populations make them even more interesting for conservation [97]. However, while the proportion of unique alleles and the genetic variation *between* populations was high in the populations from central Vietnam, we also found a high genetic variation *within* populations and a low level of inbreeding. This might be explained by the fact that northern Indo-Burma was not entirely covered by ice-sheets during the Pleistocene and this area might have served as a long-term climatically stable refugia [98,99]. Moreover, the Hoang Lien Son mountain range is a north-south oriented extension of the Himalayas with a heterogeneous topography, allowing populations to easily adapt to climate fluctuations via small shifts in their altitudinal and latitudinal range. North to south migrations during cooling and warming events are believed to be much more difficult in east-west oriented mountain ranges, making the local extinction of populations and the associated loss of genetic variation much more likely [97,100]. Another possibility is that these populations were closely connected to other, unsampled populations, especially in floristically similar regions such as northern Laos, which may have increased their genetic variation via gene flow [93].

It is clear that these populations at the southern edge of the native distribution area, together with those from northern Vietnam, contain unique and new genetic variation potentially interesting for crop improvement. Not only should more germplasm be collected from these regions, other parts of the northern Indo-Burma region (e.g. northern Laos and eastern Myanmar) should be studied as well, as they are likely suitable for *Musa balbisiana*.

## Conclusions

For the first time, Vietnamese populations of *Musa balbisiana* were genetically assessed and considerable differences between sampled populations were identified. Populations in central Vietnam, as well as a subset of populations in northern Vietnam, exhibited relatively high levels of genetic diversity comparable to native populations in China. Interestingly, Vietnamese populations showed no pattern of isolation linked to distance, indicating either excessive gene flow homogenising variation among populations or low gene flow between populations due to geographical barriers, limited long-distance pollination, and the ability to easily establish a new population after colonisation. We propose that combined clonal and sexual propagation are drivers of the observed patterns of genetic variation and structure, together with the mountain and river systems in northern Vietnam. Two populations sampled in more southern regions of Vietnam were considerably less diverse and were most likely introduced. We suggest a broader sampling of *Musa balbisiana*, as many regions with a similar suitable climate have not been investigated yet as well as neighbouring countries also in northern Indo-Burma (e.g. Laos, Myanmar). Moreover, a large scale phylogeographic study including populations from the multiple countries of the natural distribution range of *M*. *balbisiana* and in regions where *M*. *balbisiana* was introduced is necessary to further understand how this species has evolved and to what extent this was caused by human interference.

## Supporting information

S1 Fig 3D AFC of SSR genotyping data. The marked population was clearly an outlier and was removed from the study.
(TIF)

S2 Fig Chromosome tracks showing the genomic location of the 16 nuclear SSR markers retrieved from Wang et al. (2011) [64] and Rotchanapreeda et al. (2016) [65] in the reference genome sequence from a doubled-haploid accession of *Musa balbisiana* ‘Pisang Klutuk Wulung’ (DH-PKW). Marker locations are labeled in blue by the marker ID.
(TIF)

S3 Fig Results of the AMOVA for all included populations. Within population variance (Within Pops), among population variance (Among Pops), and variance among regions are presented. Regions include Northern Vietnam, Southern Vietnam, Central Vietnam, Western China, Southern China, and Hainan.
(TIF)

S4 Fig Principal coordinate analysis of wild populations of *Musa balbisiana* on the individual level based on a Euclidean distance matrix. Each individual was coloured by population.
(TIF)

S5 Fig Output of StructureSelector for determining the optimal number of K. The optimal number of K was determined for the full dataset (panels at the left hand side) and for native Vietnamese populations (panels at the right hand side): (A) MedMed K and MaxMed K through the Puechmaille method; (B), ΔK/K; (C), LnP(K)/K with K the number of clusters assumed.
(TIF)

S1 Table Overview of the 18 microsatellite markers used in this study. The underlined part of the reverse sequences indicates the sequence of the primer tail: Q1 = TGTAAAACGACGGCCAGT; Q2 = TAGGAGTGCAGCAAGCAT; Q3 = CACTGCTTAGAGCGATGC (Schuelke, 2000).
(DOCX)

S2 Table Genomic coordinates of SSR markers. 16 nuclear markers with the corresponding GenBank accession number of each sequence obtained from Wang et al. (2011) [64] and Rotchanapreeda et al. (2016) [65] together with the genomic coordinates of their single best BLAST hit.
(DOCX)

S3 Table Determination of the optimal number of clusters. The optimal number of clusters K was determined using MedMedK and MaxMedK according to the Puechmaille method, ΔK/K, and Mean LnP(K)/K. The number of clusters that were chosen for consecutive analyses are indicated in bold.
(DOCX)

## References

[1] Hajjar R, Hodgkin T. The use of wild relatives in crop improvement: a survey of developments over the last 20 years. Euphytica. 2007;156: 1–13. doi: 10.1007/s10681-007-9363-0

[2] Ray DK, Mueller ND, West PC, Foley JA. Yield Trends Are Insufficient to Double Global Crop Production by 2050. PLoS One. 2013;8. doi: 10.1371/journal.pone.0066428 23840465 PMC3686737

[3] Tilman D, Cassman KG, Matson PA, Naylor R, Polasky S. Agricultural sustainability and intensive production practices. Nature. 2002;418: 671–677. doi: 10.1038/nature01014 12167873

[4] Onyekachi OG, Boniface OO, Gemlack NF, Nicholas N. The effect of climate change on abiotic plant stress: a review. In: de Oliveira AB, editor. Abiotic and Biotic Stress in Plants. IntechOpen; 2019. p. 13. doi: 10.5772/intechopen.82681

[5] Searchinger T, Waite R, Hanson C, Ranganathan J. Creating a sustainable food future: A menu of solutions to feed nearly 10 billion people by 2050. Matthews E, editor. World Resources Report. 2019.

[6] Díez MJ, De la Rosa L, Martín I, Guasch L, Cartea ME, Mallor C, et al. Plant genebanks: Present situation and proposals for their improvement. The case of the Spanish network. Front Plant Sci. 2018;871: 1–13. doi: 10.3389/fpls.2018.01794 30564263 PMC6288731

[7] Castañeda-Álvarez NP, Khoury CK, Achicanoy HA, Bernau V, Dempewolf H, Eastwood RJ, et al. Global conservation priorities for crop wild relatives. Nat Plants. 2016;2: 1–6. doi: 10.1038/nplants.2016.22 27249561

[8] FAO. FAOSTAT Database. Rome, Italy: Food and Agriculture Organization of the United Nations; 2019 [cited 15 Feb 2021]. Available: http://www.fao.org/faostat/en/#home.

[9] FAO. Banana Market Review: Preliminary results for 2018. In: Food and Agriculture Organization of the United Nations [Internet]. Rome; 2018 p. 12. Available: http://www.fao.org/fileadmin/templates/est/COMM_MARKETS_MONITORING/Bananas/Documents/Banana_Market_Review_Prelim_Results_2018.pdf.

[10] Price NS. The origin and development of banana and plantain cultivation. In: Gowen S, editor. Bananas and Plantains. Dordrecht: Springer Netherlands; 1995. pp. 1–13. doi: 10.1007/978-94-011-0737-2_1

[11] Arias P, Dankers C, Liu P, Pilkauskas P. The World Banana Economy, 1985–2002. Rome: Food and Agriculture Organization of the United Nations; 2003. Available: http://www.fao.org/3/y5102e/y5102e00.htm.

[12] Calberto G, Staver C, Siles P. An assessment of global banana production and suitability under climate change scenarios. In: Elbehri A, editor. Climate Change and Food Systems: Global Assessments and Implications for Food Security and Trade. Rome: Food and Agriculture Organization of the United Nations; 2015. pp. 265–291.

[13] Jouneghani RS, Castro AHF, Kumar KPS, Swennen R, Luyten W. Antimicrobial activity of selected banana cultivars against important human pathogens, including candida biofilm. Foods. 2020;9: 435. doi: 10.3390/foods9040435 32260420 PMC7230924

[14] Kumar KPS, Bhowmik D, Duraivel S, Umadevi M. Traditional and medicinal uses of banana. J Pharmacogn Phytochem. 2012;1: 51–63.

[15] Kumar KPS, Castro AHF, Jouneghani RS, Leyssen P, Neyts J, Swennen R, et al. Antiviral and cytotoxic activity of different plant parts of banana (*Musa* spp.). Viruses. 2020;12. doi: 10.3390/v12050549 32429324 PMC7291111

[16] Shepherd K. Cytogenetics of the genus Musa. Montpellier, France: International Network for the Improvement of Banana and Plantain; 1999. Available: http://musalit.inibap.org/pdf/IN990087_en.pdf.

[17] Valmayor RV, Jamaluddin SH, Silayoi B, Danh LD, Pascua OC, Espino RRC. Banana cultivar names and synonyms in southeast asia. Int Netw Improv Banan Plantain—Asia Pacific. 2000; 28. Available: http://www.bioversityinternational.org/uploads/tx_news/Banana_cultivar_names_and_synonyms_in_Southeast_Asia_713.pdf.

[18] Perrier X, De Langhe E, Donohue M, Lentfer C, Vrydaghs L, Bakry F, et al. Multidisciplinary perspectives on banana (*Musa* spp.) domestication. Proc Natl Acad Sci. 2011;108: 11311–11318. doi: 10.1073/pnas.1102001108 21730145 PMC3136277

[19] Sardos J, Perrier X, Doležel J, Hřibová E, Christelová P, Van Den Houwe I, et al. DArT whole genome profiling provides insights on the evolution and taxonomy of edible Banana (*Musa* spp.). Ann Bot. 2016;118: 1269–1278. doi: 10.1093/aob/mcw170 27590334 PMC5155597

[20] Ploetz RC, Kepler AK, Daniells J, Nelson SC. Banana and plantain—an overview with emphasis on Pacific island cultivars, ver. 1. In: Elevitch CR, editor. Species Profiles for Pacific Island Agroforestry. Permanent Agriculture Resources (PAR), Hōlualoa, Hawai‘i; 2007. Available: http://traditionaltree.org.

[21] Carreel F, Fauré S, de León DG, Lagoda P, Perrier X, Bakry F, et al. Evaluation de la diversité génétique chez les bananiers diploïdes (*Musa* sp). Genet Sel Evol. 1994;26: 125s–136s. doi: 10.1186/1297-9686-26-S1-S125

[22] Němečková A, Christelová P, Čížková J, Nyine M, Van den houwe I, Svačina R, et al. Molecular and cytogenetic study of east african highland banana. Front Plant Sci. 2018;9: 1–13. doi: 10.3389/fpls.2018.00001 30337933 PMC6180188

[23] Hippolyte I, Jenny C, Gardes L, Bakry F, Rivallan R, Pomies V, et al. Foundation characteristics of edible *Musa* triploids revealed from allelic distribution of SSR markers. Ann Bot. 2012;109: 937–951. doi: 10.1093/aob/mcs010 22323428 PMC3310492

[24] Häkkinen M. Reappraisal of sectional taxonomy in *Musa* (Musaceae). Taxon. 2013;62: 809–813. doi: 10.12705/624.3

[25] Janssens SB, Vandelook F, De Langhe E, Verstraete B, Smets E, Vandenhouwe I, et al. Evolutionary dynamics and biogeography of Musaceae reveal a correlation between the diversification of the banana family and the geological and climatic history of Southeast Asia. New Phytol. 2016;210: 1453–1465. doi: 10.1111/nph.13856 26832306 PMC5066818

[26] De Langhe E, Perrier X, Donohue M, Denham T. The original banana split: Multi-disciplinary implications of the generation of African and Pacific plantains in Island Southeast Asia. Ethnobot Res Appl. 2015;14: 299–312. doi: 10.17348/era.14.0.299–312

[27] Vézina A, Turner DW, Gibbs J. Banana Sucker. 2020 [cited 12 Nov 2020]. Available: https://www.promusa.org/Banana+sucker.

[28] Argent GCG. Wild bananas of Papua New Guinea [*Ensete*, *Musa*, new taxa]. Notes from R Bot Gard Edinburgh. 1976;35: 77–114.

[29] Ge XJ, Liu MH, Wang WK, Schaal BA, Chiang TY. Population structure of wild bananas, *Musa balbisiana*, in China determined by SSR fingerprinting and cpDNA PCR-RFLP. Mol Ecol. 2005;14: 933–944. doi: 10.1111/j.1365-294X.2005.02467.x 15773926

[30] OECD. Safety Assessment of Transgenic Organisms, Volume 4: OECD Consensus Documents. Section 2. Bananas and Plantains (*Musa* spp.). Harmonisation of Regulatory Oversight in Biotechnology. Paris: OECD Publishing; 2010. pp. 84–147. doi: 10.1079/9781845936587.0000

[31] Simmonds NW, Shepherd K. The taxonomy and origins of the cultivated bananas. J Linn Soc London, Bot. 1955;55: 302–312. doi: 10.1111/j.1095-8339.1955.tb00015.x

[32] Daniells J, Jenny C, Karamura D, Tomekpe K. Musalogue: a catalogue of *Musa* germplasm. Diversity in the genus *Musa* (E. Arnaud and S. Sharrock, compil.). Montpellier, France: International Network for the Improvement of Banana and Plantain; 2001.

[33] De Langhe E, Hřibová E, Carpentier S, Dolezel J, Swennen R. Did backcrossing contribute to the origin of hybrid edible bananas? Ann Bot. 2010;106: 849–857. doi: 10.1093/aob/mcq187 20858591 PMC2990659

[34] Cenci A, Hueber Y, Zorrilla-Fontanesi Y, Van Wesemael J, Kissel E, Gislard M, et al. Effect of paleopolyploidy and allopolyploidy on gene expression in banana. BMC Genomics. 2019;20: 1–12. doi: 10.1186/s12864-018-5379-1 30917780 PMC6438041

[35] D’Hont A, Paget-Goy A, Escoute J, Carreel F. The interspecific genome structure of cultivated banana, *Musa* spp. revealed by GISH. Theor Appl Genet. 2000;100: 177–183.

[36] Baurens FC, Martin G, Hervouet C, Salmon F, Yohomé D, Ricci S, et al. Recombination and large structural variations shape interspecific edible bananas genomes. Mol Biol Evol. 2019;36: 97–111. doi: 10.1093/molbev/msy199 30403808 PMC6340459

[37] Nelson SC, Ploetz RC, Kepler AK. *Musa* species (banana and plantain), ver. 2.2. In: Elevitch CR, editor. Species Profiles for Pacific Island Agroforestry. Permanent Agriculture Resources (PAR), Hōlualoa, Hawai‘i; 2006. pp. 1–33.

[38] Vanhove AC, Vermaelen W, Panis B, Swennen R, Carpentier SC. Screening the banana biodiversity for drought tolerance: Can an in vitro growth model and proteomics be used as a tool to discover tolerant varieties and understand homeostasis. Front Plant Sci. 2012;3: 1–10. doi: 10.3389/fpls.2012.00001 22876254 PMC3410380

[39] Mattos-Moreira LA, Ferreira CF, Amorim EP, Pirovani CP, de Andrade EM, Filho MAC, et al. Differentially expressed proteins associated with drought tolerance in bananas (*Musa* spp.). Acta Physiol Plant. 2018;40: 60. doi: 10.1007/s11738-018-2638-3

[40] Zhang Q, Zhang JZ, Chow WS, Sun LL, Chen JW, Chen YJ, et al. The influence of low temperature on photosynthesis and antioxidant enzymes in sensitive banana and tolerant plantain (*Musa* sp.) cultivars. Photosynthetica. 2011;49: 201–208. doi: 10.1007/s11099-011-0012-4

[41] Tripathi L, Odipio J, Tripathi JN, Tusiime G. A rapid technique for screening banana cultivars for resistance to *Xanthomonas* wilt. Eur J Plant Pathol. 2008;121: 9–19. doi: 10.1007/s10658-007-9235-4

[42] Tripathi L, Tripathi JN, Shah T, Muiruri KS, Katari M. Molecular basis of disease resistance in banana progenitor *Musa balbisiana* against *Xanthomonas campestris* pv. *musacearum*. Sci Rep. 2019;9: 7007. doi: 10.1038/s41598-019-43421-1 31065041 PMC6504851

[43] Nakato G V., Christelová P, Were E, Nyine M, Coutinho TA, Doležel J, et al. Sources of resistance in *Musa* to *Xanthomonas campestris* pv. *musacearum*, the causal agent of banana xanthomonas wilt. Plant Pathol. 2019;68: 49–59. doi: 10.1111/ppa.12945

[44] Ocan D, Mukasa HH, Rubaihayo PR, Tinzaara W, Blomme G. Effects of banana weevil damage on plant growth and yield of East African Musa genotypes. J Appl Biosci. 2008;9: 407–415.

[45] Wang Z, Miao H, Liu J, Xu B, Yao X, Xu C, et al. *Musa balbisiana* genome reveals subgenome evolution and functional divergence. Nat Plants. 2019. doi: 10.1038/s41477-019-0452-6 31308504 PMC6784884

[46] Bawin Y, Panis B, Vanden Abeele S, Li Z, Sardos J, Paofa J, et al. Genetic diversity and core subset selection in ex situ seed collections of the banana crop wild relative *Musa balbisiana*. Plant Genet Resour Characterisation Util. 2019;17: 536–544. doi: 10.1017/S1479262119000376

[47] Eyland D, Breton C, Sardos J, Kallow S, Panis B, Swennen R, et al. Filling the gaps in gene banks: Collecting, characterizing, and phenotyping wild banana relatives of Papua New Guinea. Crop Sci. 2021;61: 137–149. doi: 10.1002/csc2.20320

[48] Uma S, Siva SA, Saraswathi MS, Manickavasagam M, Durai P, Selvarajan R, et al. Variation and intraspecific relationships in Indian wild *Musa balbisiana* (BB) population as evidenced by random amplified polymorphic DNA. Genet Resour Crop Evol. 2006;53: 349–355. doi: 10.1007/s10722-004-0576-y

[49] Wang X-L, Chiang T-Y, Roux N, Hao G, Ge X-J. Genetic diversity of wild banana (*Musa balbisiana* Colla) in China as revealed by AFLP markers. Genet Resour Crop Evol. 2007;54: 1125–1132. doi: 10.1007/s10722-006-9004-9

[50] Ford-Lloyd B V., Schmidt M, Armstrong SJ, Barazani O, Engels J, Hadas R, et al. Crop wild relatives—undervalued, underutilized and under threat? Bioscience. 2011;61: 559–565. doi: 10.1525/bio.2011.61.7.10

[51] Vincent H, Amri A, Castañeda-Álvarez NP, Dempewolf H, Dulloo E, Guarino L, et al. Modeling of crop wild relative species identifies areas globally for in situ conservation. Commun Biol. 2019;2: 1–8. doi: 10.1038/s42003-018-0242-0 31044161 PMC6478866

[52] Mertens A, Swennen R, Rønsted N, Vandelook F, Panis B, Sachter-Smith G, et al. Conservation status assessment of banana crop wild relatives using species distribution modelling. Divers Distrib. 2021; 1–18. 10.1111/ddi.13233.

[53] Brown A, Tumuhimbise R, Amah D, Uwimana B, Nyine M, Mduma H, et al. Bananas and plantains (*Musa* spp.). Genetic Improvement of Tropical Crops. Cham: Springer; 2017. pp. 219–240. doi: 10.1007/978-3-319-59819-2_7

[54] Ude G, Pillay M, Nwakanma D, Tenkouano A. Genetic Diversity in *Musa acuminata* Colla and *Musa balbisian*a Colla and some of their natural hybrids using AFLP Markers. Theor Appl Genet. 2002;104: 1246–1252. doi: 10.1007/s00122-002-0914-4 12582577

[55] Eckert GC, Samis KE, Lougheed SC. Genetic variation across species’ geographical ranges: the central–marginal hypothesis and beyond. Mol Ecol. 2008;17: 1170–1188. Available: doi: 10.1111/j.1365-294X.2007.03659.x 18302683

[56] De Kort H, Prunier JG, Ducatez S, Honnay O, Baguette M, Stevens VM, et al. Life history, climate and biogeography interactively affect worldwide genetic diversity of plant and animal populations. Nat Commun. 2021;12. doi: 10.1038/s41467-020-20168-2 33483517 PMC7822833

[57] Garner TWJ, Pearman PB, Angelone S. Genetic diversity across a vertebrate species’ range: A test of the central-peripheral hypothesis. Mol Ecol. 2004;13: 1047–1053. doi: 10.1111/j.1365-294X.2004.02119.x 15078443

[58] Brown JH. On the Relationship between Abundance and Distribution of Species. Am Nat. 1984;124: 255–279. doi: 10.1086/284267

[59] The World Bank. World Bank Official Boundaries. 2020. Available: https://datacatalog.worldbank.org/dataset/world-bank-official-boundaries.

[60] The World Bank. World—Terrain Elevation Above Sea Level (ELE) GIS Data, (Global Solar Atlas), Global Solar Atlas. 2020. Available: https://datacatalog.worldbank.org/dataset/world-terrain-elevation-above-sea-level-ele-gis-data-global-solar-atlas.

[61] Dinerstein E, Olson D, Joshi A, Vynne C, Burgess ND, Wikramanayake E, et al. An ecoregion-based approach to protecting half the terrestrial realm. Bioscience. 2017;67: 534–545. doi: 10.1093/biosci/bix014 28608869 PMC5451287

[62] Chase MW, Hills HH. Silica gel: An ideal material for field preservation of leaf samples for DNA studies. Taxon. 1991;40: 215–220. 10.2307/1222975.

[63] Doyle JJ, Doyle JL. A rapid DNA isolation procedure for small quantities of fresh leaf tissue. Phytochem Bull. 1987;19: 11–15.

[64] Wang JY, Huang BZ, Chen YY, Feng SP, Wu YT. Identification and characterization of microsatellite markers from *Musa balbisiana*. Plant Breed. 2011;130: 584–590. doi: 10.1111/j.1439-0523.2011.01861.x

[65] Rotchanapreeda T, Wongniam S, Swangpol SC, Chareonsap PP, Sukkaewmanee N, Somana J. Development of SSR markers from *Musa balbisiana* for genetic diversity analysis among Thai bananas. Plant Syst Evol. 2016;302: 739–761. doi: 10.1007/s00606-015-1274-2

[66] Droc G, Larivière D, Guignon V, Yahiaoui N, This D, Garsmeur O, et al. The banana genome hub. Database. 2013;2013: 1–14. doi: 10.1093/database/bat035 23707967 PMC3662865

[67] R Core Team. R: A language and environment for statistical computing. Vienna, Austria: R Foundation for Statistical Computing; 2020. Available: https://www.r-project.org/.

[68] Belkhir K, Borsa P, Chikhi L, Raufaste N, Bonhomme F. GENETIX 4.05, logiciel sous Windows TM pour la génétique des populations. Montpellier, France: Université de Montpellier II; 2004. doi: 10.1159/000078211

[69] Peakall R, Smouse PE. GenAlEx 6.5: genetic analysis in Excel. Population genetic software for teaching and research—an update. Bioinformatics. 2012;28: 2537–2539. Available: doi: 10.1093/bioinformatics/bts460 22820204 PMC3463245

[70] Goudet J. hierfstat, a package for r to compute and test hierarchical F-statistics. Mol Ecol Notes. 2005;5: 184–186. 10.1111/j.1471-8286.2004.00828.x.

[71] Dray S, Dufour A-B. The ade4 package: implementing the duality diagram for ecologists. J Stat Softw. 2007;22: 1–20. doi: 10.18637/jss.v022.i04

[72] Pritchard JK, Stephens M, Donnelly P. Inference of population structure using multilocus genotype data. Genetics. 2000;155: 945–59. Available: http://www.ncbi.nlm.nih.gov/pubmed/10835412. 10835412 10.1093/genetics/155.2.945 PMC1461096

[73] Afgan E, Baker D, van den Beek M, Blankenberg D, Bouvier D, Čech M, et al. The Galaxy platform for accessible, reproducible and collaborative biomedical analyses: 2016 update. Nucleic Acids Res. 2016;44: W3–W10. doi: 10.1093/nar/gkw343 27137889 PMC4987906

[74] Wang J. The computer program structure for assigning individuals to populations: easy to use but easier to misuse. Mol Ecol Resour. 2017;17: 981–990. doi: 10.1111/1755-0998.12650 28028941

[75] Evanno G, Regnaut S, Goudet J. Detecting the number of clusters of individuals using the software STRUCTURE: A simulation study. Mol Ecol. 2005;14: 2611–2620. doi: 10.1111/j.1365-294X.2005.02553.x 15969739

[76] Puechmaille SJ. The program structure does not reliably recover the correct population structure when sampling is uneven: subsampling and new estimators alleviate the problem. Mol Ecol Resour. 2016;16: 608–627. doi: 10.1111/1755-0998.12512 26856252

[77] Li YL, Liu JX. StructureSelector: A web-based software to select and visualize the optimal number of clusters using multiple methods. Mol Ecol Resour. 2018;18: 176–177. doi: 10.1111/1755-0998.12719 28921901

[78] Kopelman NM, Mayzel J, Jakobsson M, Rosenberg NA, Mayrose I. Clumpak: a program for identifying clustering modes and packaging population structure inferences across K. Mol Ecol Resour. 2015;15: 1179–1191. doi: 10.1111/1755-0998.12387 25684545 PMC4534335

[79] Janes JK, Miller JM, Dupuis JR, Malenfant RM, Gorrell JC, Cullingham CI, et al. The K = 2 conundrum. Mol Ecol. 2017;26: 3594–3602. doi: 10.1111/mec.14187 28544181

[80] Herden T, Bönisch M, Friesen N. Genetic diversity of *Helosciadium repens* (Jacq.) W.D.J. Koch (Apiaceae) in Germany, a Crop Wild Relative of celery. Ecol Evol. 2019;10: 875–890. doi: 10.1002/ece3.5947 32015851 PMC6988547

[81] Olsen KM, Schaal BA. Microsatellite variation in cassava (*Manihot esculenta*, Euphorbiaceae) and its wild relatives: Further evidence for a southern Amazonian origin of domestication. Am J Bot. 2001;88: 131–142. doi: 10.2307/2657133 11159133

[82] Rodriguez-Bonilla L, Williams KA, Rodríguez Bonilla F, Matusinec D, Maule A, Coe K, et al. The genetic diversity of cranberry crop wild relatives, *Vaccinium macrocarpon* Aiton and *V. Oxycoccos* L., in the US, with special emphasis on national forests. Plants. 2020;9: 1–22. doi: 10.3390/plants9111446 33114692 PMC7716231

[83] Cole CT. Genetic Variation in rare and common plants. Annu Rev Ecol Evol Syst. 2003;34: 213–237. doi: 10.1146/annurev.ecolsys.34.030102.151717

[84] Bona A, Kulesza U, Jadwiszczak KA. Clonal diversity, gene flow and seed production in endangered populations of *Betula humilis* Schrk. Tree Genet Genomes. 2019;15. doi: 10.1007/s11295-019-1357-2

[85] Navascués M, Stoeckel S, Mariette S. Genetic diversity and fitness in small populations of partially asexual, self-incompatible plants. Heredity (Edinb). 2009/11/18. 2010;104: 482–492. doi: 10.1038/hdy.2009.159 19920857 PMC3358671

[86] Pappert RA, Hamrick JL, Donovan LA. Genetic variation in *Pueraria lobata* (Fabaceae), an introduced, clonal, invasive plant of the southeastern United States. Am J Bot. 2000;87: 1240–1245. doi: 10.2307/2656716 10991894

[87] Stoeckel S, Grange J, Fernández-Manjarres JF, Bilger I, Frascaria-Lacoste N, Mariette S. Heterozygote excess in a self-incompatible and partially clonal forest tree species—*Prunus avium* L. Mol Ecol. 2006;15: 2109–2118. doi: 10.1111/j.1365-294X.2006.02926.x 16780428

[88] Kruskop S V. Bats of Vietnam: Checklist and an identification manual. 2nd ed. Korzun LP, Kalyakin MV, editors. Moscow: KMK Scientific Press; 2013.

[89] Thanh HT, Son NT, Duong VT, Luong NT, Loi DN, Thong VD. New records and morphological assessments of long-nosed fruit bats (chiroptera: pteropodidae: *Macroglossus* spp.) from Vietnam. Tap Chi Sinh Hoc. 2019;41: 117–124. doi: 10.15625/0866-7160/v41n4.14695

[90] Frick WF, Kingston T, Flanders J. A review of the major threats and challenges to global bat conservation. Ann N Y Acad Sci. 2020;1469: 5–25. doi: 10.1111/nyas.14045 30937915

[91] Yuan ZY, Suwannapoom C, Yan F, Poyarkov NA, Nguyen SN, Chen HM, et al. Red River barrier and Pleistocene climatic fluctuations shaped the genetic structure of *Microhyla fissipes* complex (Anura: Microhylidae) in southern China and Indochina. Curr Zool. 2016;62: 531–543. doi: 10.1093/cz/zow042 29491943 PMC5804247

[92] Yang R, Feng X, Gong X. Genetic structure and demographic history of *Cycas chenii* (Cycadaceae), an endangered species with extremely small populations. Plant Divers. 2017;39: 44–51. doi: 10.1016/j.pld.2016.11.003 30159490 PMC6112254

[93] Averyanov L V, Loc PK, Hiep NT, Harder DK. Phytogeographic review of Vietnam and adjacent areas of Eastern Indochina. Komarovia. 2003;3: 1–83.

[94] Balloux F, Lehmann L, De Meeûs T. The population genetics of clonal and partially clonal diploids. Genetics. 2003;164: 1635–1644. doi: 10.2135/cropsci1967.0011183X000700040005 12930767 PMC1462666

[95] Start AN, Marshall AG. Nectarivorous bats as pollinators of trees in West Malaysia. In: Burley K, Styles BT, editors. Tropical Trees: Variation, Breeding and Conservation. London: Academic Press; 1976. pp. 141–149.

[96] Lefebvre V, Villemant C, Fontaine C, Daugeron C. Altitudinal, temporal and trophic partitioning of flower-visitors in Alpine communities. Sci Rep. 2018;8: 1–12. doi: 10.1038/s41598-017-17765-5 29549294 PMC5856740

[97] Hampe A, Petit RJ. Conserving biodiversity under climate change: The rear edge matters. Ecol Lett. 2005;8: 461–467. doi: 10.1111/j.1461-0248.2005.00739.x 21352449

[98] Ehlers J, Gibbard PL. The extent and chronology of Cenozoic Global Glaciation. Quat Int. 2007;164–165: 6–20. doi: 10.1016/j.quaint.2006.10.008

[99] Tang CQ, Matsui T, Ohashi H, Dong YF, Momohara A, Herrando-Moraira S, et al. Identifying long-term stable refugia for relict plant species in East Asia. Nat Commun. 2018;9. doi: 10.1038/s41467-017-01881-x 30367062 PMC6203703

[100] Badgley C. Tectonics, topography, and mammalian diversity. Ecography (Cop). 2010;33: 220–231. 10.1111/j.1600-0587.2010.06282.x.

