# Supplementary figures and images for: Genetic diversity and structure of Musa balbisiana populations in Vietnam and its implications for the conservation of banana crop wild relatives
Source: PLoS One. 2021 Jun 23;16(6):e0253255. doi: 10.1371/journal.pone.0253255 (PMC8221469; doi:10.1371/journal.pone.0253255)

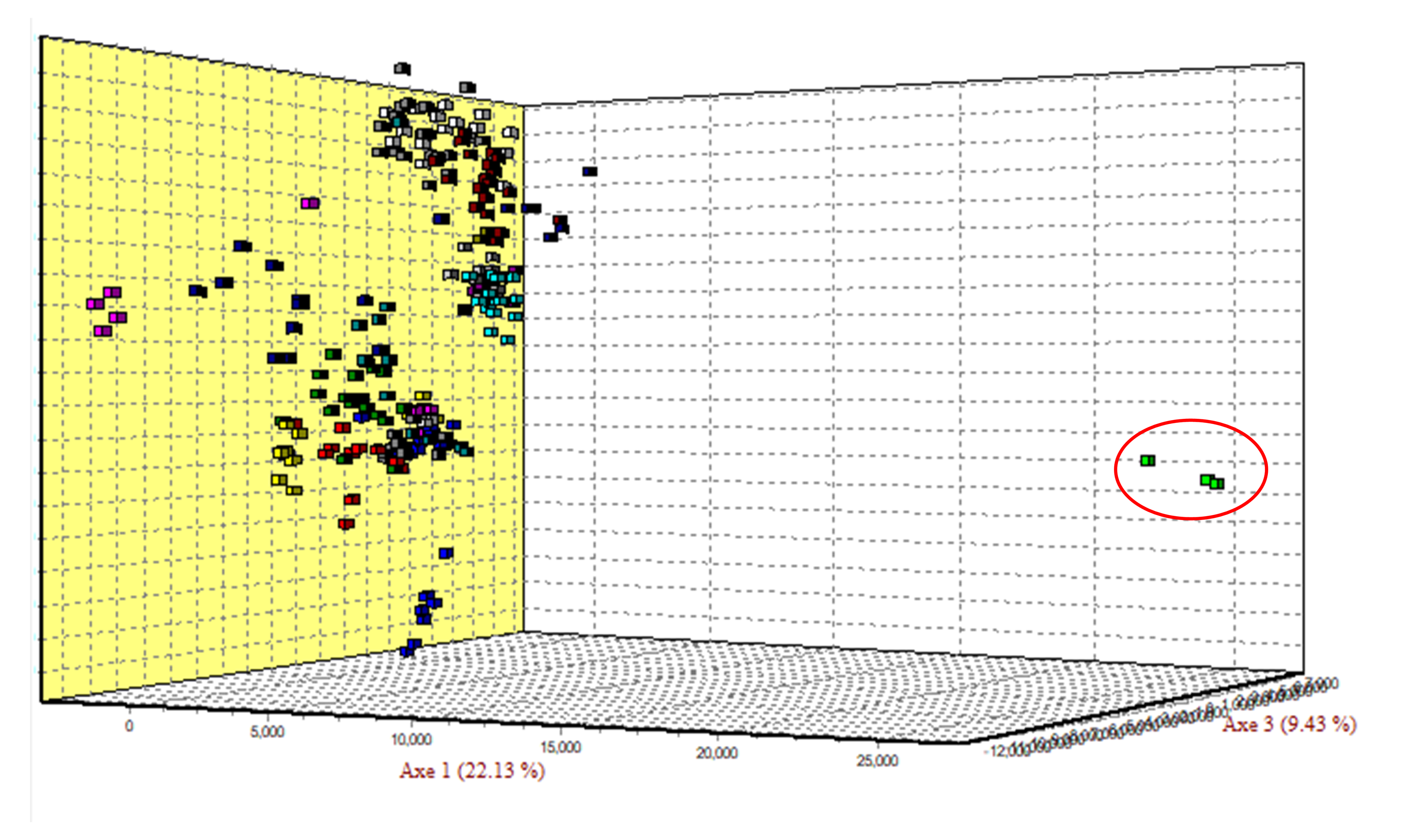

Supplement: S1 Fig — The marked population was clearly an outlier and was removed from the study. (TIF) [file pone.0253255.s001.tif]

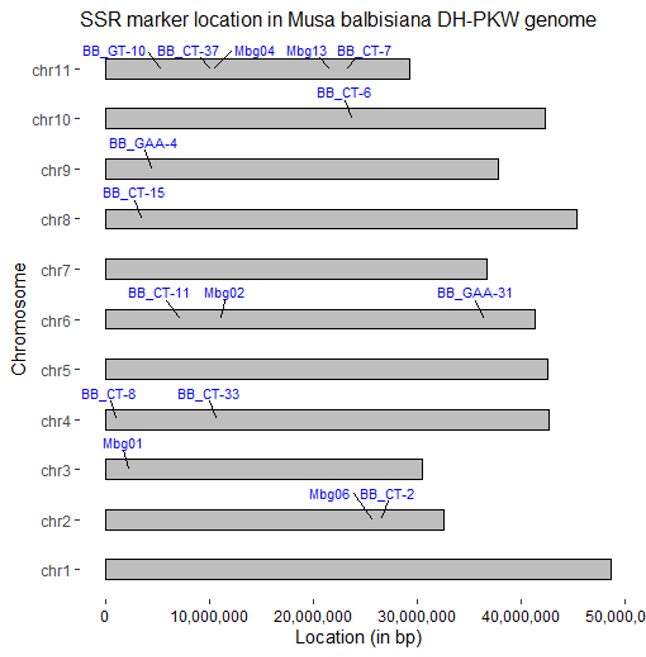

Supplement: S2 Fig — Marker locations are labeled in blue by the marker ID. (TIF) [file pone.0253255.s002.tif]

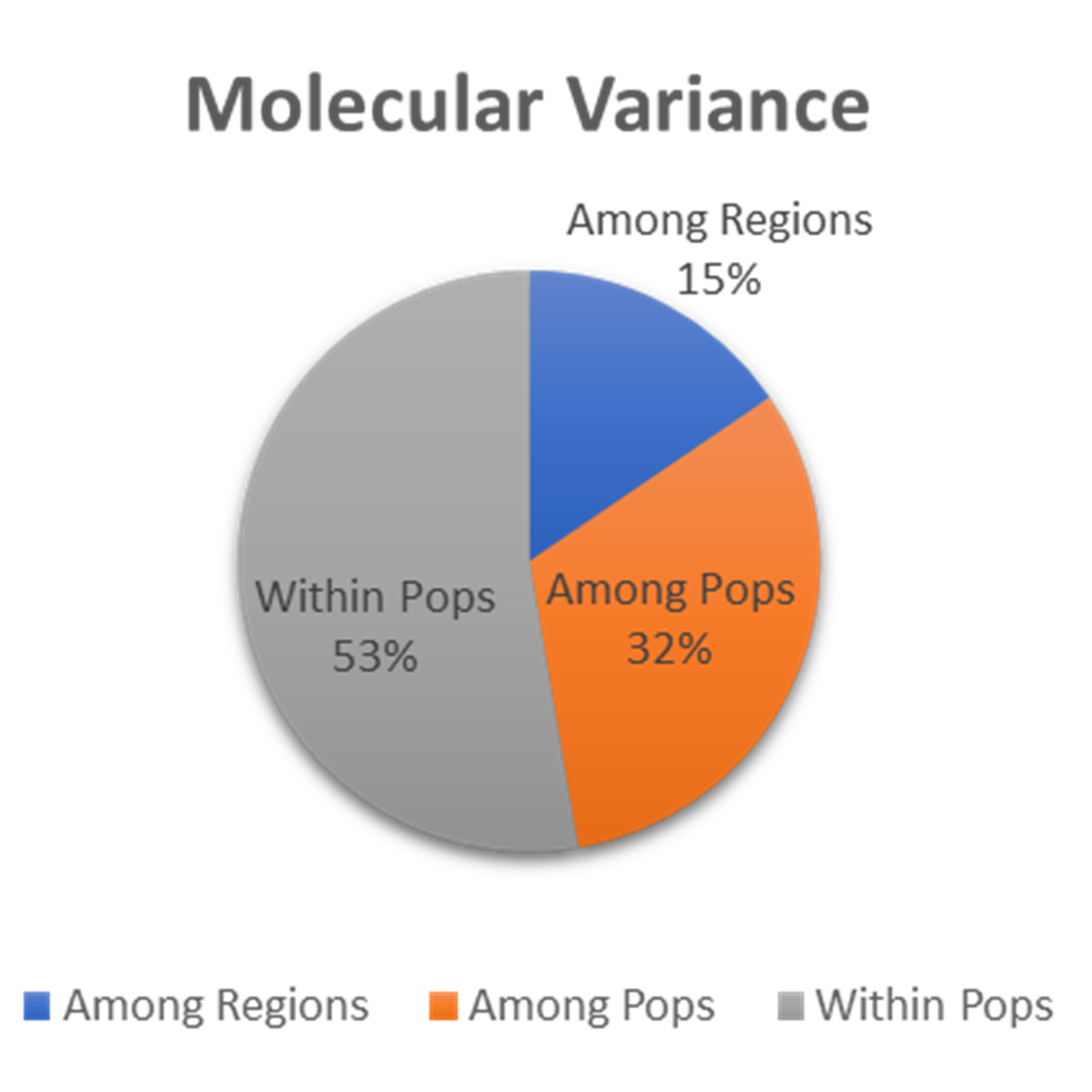

Supplement: S3 Fig — Within population variance (Within Pops), among population variance (Among Pops), and variance among regions are presented. Regions include Northern Vietnam, Southern Vietnam, Central Vietnam, Western China, Southern China, and Hainan. (TIF) [file pone.0253255.s003.tif]

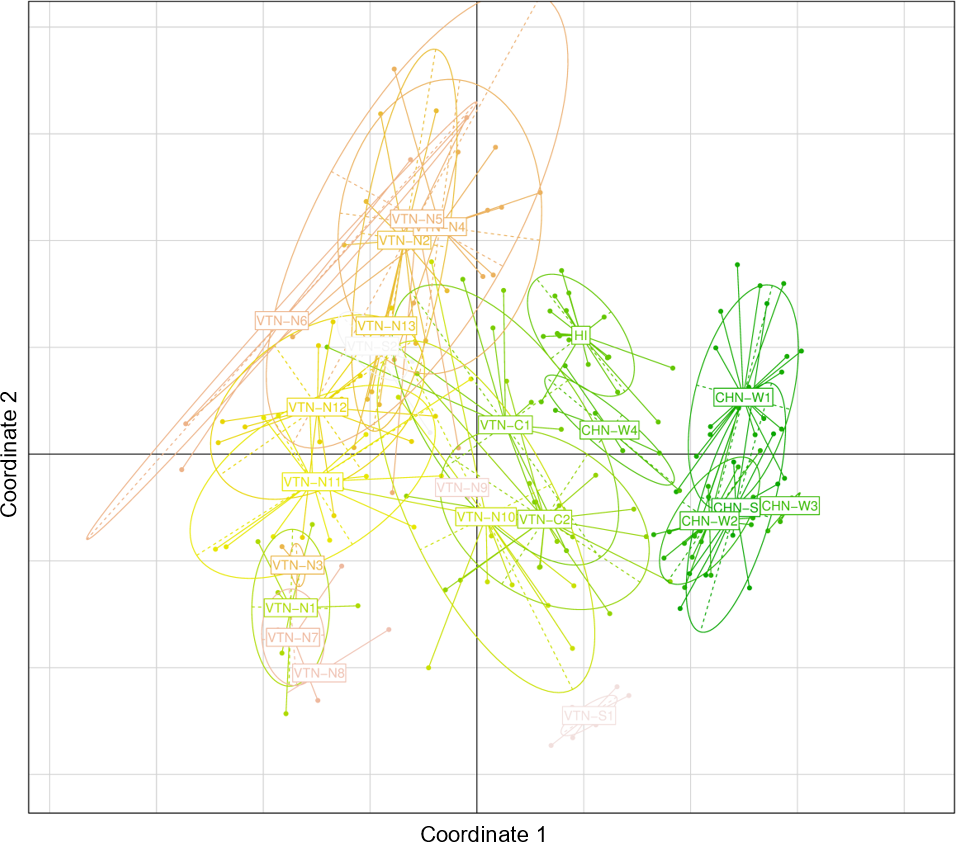

Supplement: S4 Fig — Each individual was coloured by population. (TIF) [file pone.0253255.s004.tif]

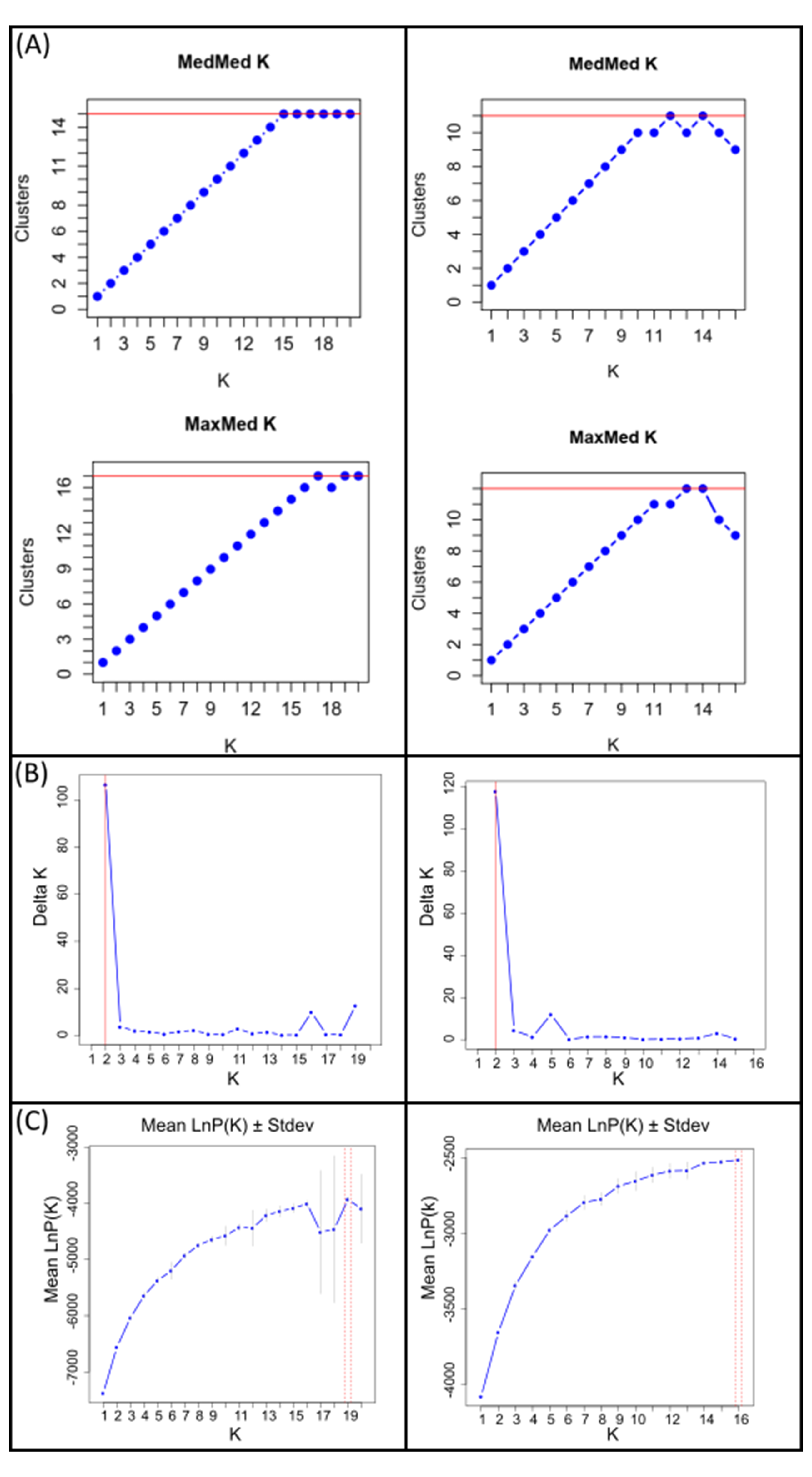

Supplement: S5 Fig — The optimal number of K was determined for the full dataset (panels at the left hand side) and for native Vietnamese populations (panels at the right hand side): (A) MedMed K and MaxMed K through the Puechmaille method; (B), ΔK/K; (C), LnP(K)/K with K the number of clusters assumed. (TIF) [file pone.0253255.s005.tif]
