# Supplementary material for: Genetic diversity and structure of Musa balbisiana populations in Vietnam and its implications for the conservation of banana crop wild relatives
Source: PLoS One. 2021 Jun 23;16(6):e0253255. doi: 10.1371/journal.pone.0253255 (PMC8221469; doi:10.1371/journal.pone.0253255)
Supplement: S1 Table — The underlined part of the reverse sequences indicates the sequence of the primer tail: Q1 = TGTAAAACGACGGCCAGT; Q2 = TAGGAGTGCAGCAAGCAT; Q3 = CACTGCTTAGAGCGATGC (Schuelke, 2000). (DOCX) [file pone.0253255.s006.docx]

| Locus | | Repeat motif | Primer sequence (5’→3’) | Labeled primer | Expected Allele size | Reference |
| --- | --- | --- | --- | --- | --- | --- |
| *Multiplex 1* |  |  |  |  |  |  |
| BB_GAA-31 | | (AG)_6_ | ACCGAAGAAAACGAAGCAGA | Q1-6-FAM | 155-432 | (Rotchanapreeda et al. 2016) |
|  | |  | TGTAAAACGACGGCCAGTGACCCTCGGATGTGTGTACC |  |  |  |
| Mbg02 | | (GA)_7_ | ACACCGAGAGAGAGAGAGAGGACAC | Q1-6-FAM | 140-260 | (Wang et al. 2011) |
|  | |  | TGTAAAACGACGGCCAGTTAGTGCGCTGATATGTCGAGTGC |  |  |  |
| Mbg04 | | (GA)_8;_(TTTGC)_4_(TG)_2_ (TA)_5_GGG (AT)_3_ | CCGAGAGAGAGAGAGAGAGGGTTTG | Q2-NED | 122-145 | (Wang et al. 2011) |
|  | |  | TAGGAGTGCAGCAAGCATCTCCATCAGTCATGTAAAGCCCC |  |  |  |
| Mbg13 | | (AGG)_3_AG(AGG) | AAGAACAGAGCCAAGTGCG | Q2-NED | 180-236 | (Wang et al. 2011) |
|  | |  | TAGGAGTGCAGCAAGCATGTCCCGTTCTTGGTAACTTTGT |  |  |  |
| BB_CT-37 | | (TG)_9_(GA)_21_ | TGTTGGATTGCCATGGTATG | Q3-VIC | 88-456 | (Rotchanapreeda et al. 2016) |
|  | |  | CACTGCTTAGAGCGATGCTCCGAGTCTACTCCCAAGGA |  |  |  |
| *Multiplex 2* |  |  |  |  |  |  |
| MaSSR01 | | Unknown | TGAGGCGGGGAATCGGTA | Q1-6-FAM | 100-150 | (Ge et al. 2005) |
|  | |  | TGTAAAACGACGGCCAGTGGCGGGAGACAGATGGAGTT |  |  |  |
| BB_CT-11 | | (TC)_8_ | GGCTGTACTCCTGTGGTGGT | Q1-6-FAM | 95-459 | (Rotchanapreeda et al. 2016) |
|  | |  | TGTAAAACGACGGCCAGTTGTCACCAATCCATGACCAG |  |  |  |
| BB_GAA-4 | | (AG)_22_(CA)_4_ | GATTGCTTGCAGATAATGAACTTT | Q2-NED | 92-236 | (Rotchanapreeda et al. 2016) |
|  | |  | TAGGAGTGCAGCAAGCATGTACCGAAGCTTCCACCAAA |  |  |  |
| BB_CT-33 | | (TG)_8_(GA)_10_ | GGCAATGTCTCATAAGAAAGAGAG | Q3-VIC | 99-239 | (Rotchanapreeda et al. 2016) |
|  | |  | CACTGCTTAGAGCGATGCTTTTGCACCTTTGCAGAGAA |  |  |  |
| *Multiplex 3* |  |  |  |  |  |  |
| Mbg06 | | (GAA)_8_ | AGCAACCCGTGGATAAAGAGC | Q1-6-FAM | 125-190 | (Wang et al. 2011) |
|  | |  | TGTAAAACGACGGCCAGTTCCCTCTCGCTCCTCTTCTTC |  |  |  |
| BB_CT-15 | | (TC)_20_ | CACCATTTGTGATGCCACTC | Q1-6-FAM | 85-244 | (Rotchanapreeda et al. 2016) |
|  | |  | TGTAAAACGACGGCCAGTTAGGCCACATACCCAGCTTC |  |  |  |
| BB_GT-10 | | (GA)_5_ | GAGTGATCCCACCTTGAGGA | Q2-NED | 109-337 | (Rotchanapreeda et al. 2016) |
|  | |  | TAGGAGTGCAGCAAGCATGCCAACCATCATTGGAGAC |  |  |  |
| BB_AAC-3 | | (TC)_4_(TC)_4_ | AAATTCGGGGGTCAAAAAGT | Q3-VIC | 157-172 | (Rotchanapreeda et al. 2016) |
|  | |  | CACTGCTTAGAGCGATGCGAGGGATTTATGGGACGACA |  |  |  |
| BB_CT-7 | | (AG)_5_ | ACGCAACGAGACACACAAAC | Q3-VIC | 109-231 | (Rotchanapreeda et al. 2016) |
|  | |  | CACTGCTTAGAGCGATGCGAACGAGAAACTGCCTTTGC |  |  |  |
| *Multiplex 4* |  |  |  |  |  |  |
| BB_CT-6 | | (TC)_19_(AC)_5_ | GGCTTGGTCATCAGAGGAAG | Q1-6-FAM | 115-288 | (Rotchanapreeda et al. 2016) |
|  | |  | TGTAAAACGACGGCCAGTTGAAGCCAAACCTTTATTGC |  |  |  |
| Mbg01 | | (GA)_6_ | GAGAGAGAGAGATCGTTTAGCAGTG | Q2-NED | 130-180 | (Wang et al. 2011) |
|  | |  | TAGGAGTGCAGCAAGCATAGAGGCTCGTGATTCATGTGGTC |  |  |  |
| BB_CT-8 | | (CT)_18_ | GTTCAAGCATCCTCAGCACA | Q2-NED | 102-274 | (Rotchanapreeda et al. 2016) |
|  | |  | TAGGAGTGCAGCAAGCATCCGAAAGGAGAAACCAGTTG |  |  |  |
| BB_CT-2 | | (AG)_10_ | TTGTTTTGCTGATGCTGACC | Q3-VIC | 123-271 | (Rotchanapreeda et al. 2016) |
|  | |  | CACTGCTTAGAGCGATGCGCGATAACATTCTCCGCAAT |  |  |  |

**S1 Table.** **Overview of the 18 microsatellite markers used in this study.**

The underlined part of the reverse sequences indicates the sequence of the primer tail: Q1 = TGTAAAACGACGGCCAGT; Q2 = TAGGAGTGCAGCAAGCAT; Q3 = CACTGCTTAGAGCGATGC (Schuelke 2000).

**References**

Ge, X. J. et al. 2005. “Population Structure of Wild Bananas, *Musa Balbisiana*, in China Determined by SSR Fingerprinting and CpDNA PCR-RFLP.” *Molecular Ecology* 14(4): 933–44.

Rotchanapreeda, Tiwa et al. 2016. “Development of SSR Markers from *Musa Balbisiana* for Genetic Diversity Analysis among Thai Bananas.” *Plant Systematics and Evolution* 302(7): 739–61.

Schuelke, M. 2000. “An Economic Method for the Fluorescent Labeling of PCR Fragments.” *Nature Biotechnology* 18(2): 233–34.

Wang, Jing Yi et al. 2011. “Identification and Characterization of Microsatellite Markers from *Musa Balbisiana*.” *Plant Breeding* 130(5): 584–90.
