# Supplementary material for: Genetic diversity and structure of Musa balbisiana populations in Vietnam and its implications for the conservation of banana crop wild relatives
Source: PLoS One. 2021 Jun 23;16(6):e0253255. doi: 10.1371/journal.pone.0253255 (PMC8221469; doi:10.1371/journal.pone.0253255)
Supplement: S2 Table — 16 nuclear markers with the corresponding GenBank accession number of each sequence obtained from Wang et al. (2011) [64] and Rotchanapreeda et al. (2016) [65] together with the genomic coordinates of their single best BLAST hit. (DOCX) [file pone.0253255.s007.docx]

**S2 Table. Genomic coordinates of SSR markers.**

| **Marker ID** | **GenBank accession number** | **Chromosome** | **Start** | **End** |
| --- | --- | --- | --- | --- |
| Mbg06 | GQ466104 | Chr2 | 25807518 | 25807781 |
| BB_CT-2 | KM190159 | Chr2 | 26502276 | 26502707 |
| Mbg01 | GQ466093 | Chr3 | 2200863 | 2201143 |
| BB_CT-8 | KM190163 | Chr4 | 1054966 | 1055508 |
| BB_CT-33 | KM190164 | Chr4 | 10700566 | 10700953 |
| BB_CT-11 | KM190156 | Chr6 | 7219627 | 7220209 |
| Mbg02 | GQ466098 | Chr6 | 11039692 | 11039879 |
| BB_GAA-31 | KM190154 | Chr6 | 36462591 | 36463133 |
| BB_CT-15 | KM190160 | Chr8 | 3481150 | 3481862 |
| BB_GAA-4 | KM190169 | Chr9 | 4464489 | 4464697 |
| BB_CT-6 | KM190174 | Chr10 | 23719714 | 23720015 |
| BB_GT-10 | KM190166 | Chr11 | 5340674 | 5341317 |
| BB_CT-37 | KM190157 | Chr11 | 10120116 | 10120530 |
| Mbg04 | GQ466100 | Chr11 | 10314001 | 10314152 |
| Mbg13 | EF467427 | Chr11 | 21584067 | 21584574 |
| BB_CT-7 | KM190149 | Chr11 | 23251219 | 23251522 |

16 nuclear markers with the corresponding GenBank accession number of each sequence obtained from (Wang et al. 2011) and (Rotchanapreeda et al. 2016) together with the genomic coordinates of their single best BLAST hit.

**References**

Rotchanapreeda, Tiwa et al. 2016. “Development of SSR Markers from *Musa Balbisiana* for Genetic Diversity Analysis among Thai Bananas.” *Plant Systematics and Evolution* 302(7): 739–61.

Wang, Jing Yi et al. 2011. “Identification and Characterization of Microsatellite Markers from *Musa Balbisiana*.” *Plant Breeding* 130(5): 584–90.
