# Supplementary material for: Genetic diversity and structure of Musa balbisiana populations in Vietnam and its implications for the conservation of banana crop wild relatives
Source: PLoS One. 2021 Jun 23;16(6):e0253255. doi: 10.1371/journal.pone.0253255 (PMC8221469; doi:10.1371/journal.pone.0253255)
Supplement: S3 Table — The optimal number of clusters K was determined using MedMedK and MaxMedK according to the Puechmaille method, ΔK/K, and Mean LnP(K)/K. The number of clusters that were chosen for consecutive analyses are indicated in bold. (DOCX) [file pone.0253255.s008.docx]

**S3 Table. Determination of the optimal number of clusters.**

|  |  | MedMedK | MaxMedK | ΔK/K | Mean LnP(K)/K |
| --- | --- | --- | --- | --- | --- |
| Optimal K | All populations | 15 | 16 | **2** or 16 | 19 |
|  | Native Vietnamese populations | 11 | 12 | 2 or **5** | 16 |

The optimal number of clusters K was determined using MedMedK and MaxMedK according to the Puechmaille method, ΔK/K, and Mean LnP(K)/K. The number of clusters that were chosen for consecutive analyses are indicated in bold.
